# Dual targeting of the cancer antioxidant network with 1,4-naphthoquinone fused Gold(i) N-heterocyclic carbene complexes†
†Electronic supplementary information (ESI) available. CCDC 1520307, 1537344. For ESI and crystallographic data in CIF or other electronic format see DOI: 10.1039/c7sc02153d


**DOI:** 10.1039/c7sc02153d

**Published:** 2017-07-21

**Authors:** R. McCall, M. Miles, P. Lascuna, B. Burney, Z. Patel, K. J. Sidoran, V. Sittaramane, J. Kocerha, D. A. Grossie, J. L. Sessler, K. Arumugam, J. F. Arambula

**Affiliations:** a Department of Chemistry , Georgia Southern University , Statesboro , GA 30460 , USA . Email: jfarambula@cm.utexas.edu; b Department of Chemistry , Wright State University , 3640 Colonel Glenn Hwy , Dayton , Ohio 45435 , USA . Email: kuppuswamy.arumugam@wright.edu; c Department of Biology , Georgia Southern University , Statesboro , GA 30460 , USA; d Department of Chemistry , St. Bonaventure University , St. Bonaventure , NY 14778 , USA; e Department of Chemistry , University of Texas , 105 E. 24th St. , Austin , TX 78712-1224 , USA

## Abstract

To achieve a systems-based approach to targeting the antioxidant pathway, 1,4-naphthoquinone annulated N-heterocyclic carbene (NHC) [bis(1,3-dimesityl-4,5-naphthoquino-imidazol-2-ylidene)-gold(i)] [silver(i) dichloride] (**1**), [bis(1,3-dimesityl-4,5-naphthoquino-imidazol-2-ylidene)-gold(i)] chloride (**2**), and 1,3-dimesityl-4,5-naphthoquino-imidazol-2-ylidene)-gold(i) chloride (**3**)) were designed, synthesized, and tested for biological activity in a series of human cancer cell lines. The solution phase of complexes **1–3** were assigned using several spectroscopy techniques, including NMR spectroscopic analysis. Complexes **1** and **3** were further characterized by single crystal X-ray diffraction analysis. Electrochemical and spectroelectrochemical studies revealed that quinone reductions are reversible and that the electrochemically generated semiquinone and quinone dianions are stable under these conditions. Complex **1**, containing two NHC-quinone moieties (to accentuate exogenous ROS *via* redox cycling) centered around a Au(i) center (to inactivate thioredoxin reductase (TrxR) irreversibly), was found to inhibit cancer cell proliferation to a much greater extent than the individual components (*i.e.*, Au(i)–NHC alone or naphthoquinone alone). Treatment of A549 lung cancer cells with **1** produced a 27-fold increase in exogenous reactive oxygen species (ROS) which was found to localize to the mitochondria. The inhibition of TrxR, an essential mediator of ROS homeostasis, was achieved in the same cell line at low administrated concentrations of **1**. TrxR inhibition by **1** was similar to that of auranofin, a gold(i) containing complex known to inhibit TrxR irreversibly. Complex **1** was found to induce cell death *via* an apoptotic mechanism as confirmed by annexin-V staining. Complex **1** was demonstrated to be efficacious in zebrafish bearing A549 xenografts. These results provide support for the suggestion that a dual targeting approach that involves reducing ROS tolerance while concurrently increasing ROS production can perturb antioxidant homeostasis, enhance cancer cell death *in vitro*, and reduce tumor burden *in vivo*, as inferred from preliminary zebra fish model studies.

## Abstract 2

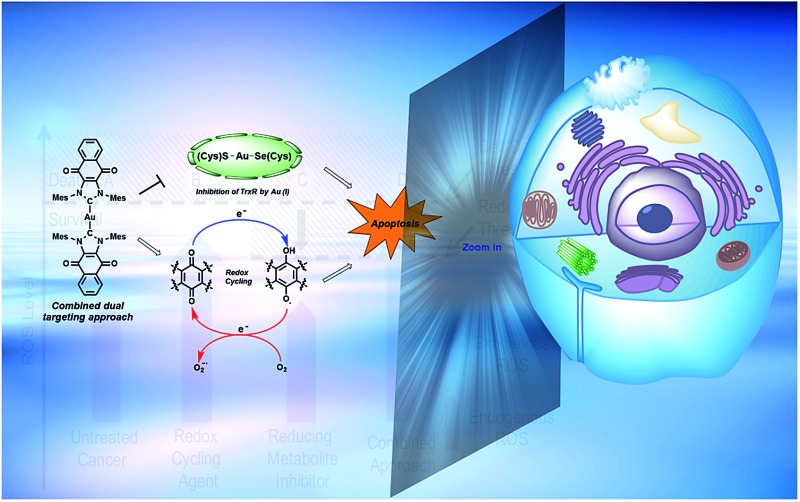
Herein, we report a simple and rational approach to the design of a targeted therapy (*i.e.*, complex **1**) whose mechanism of action involves targeting a single cancer relevant pathway *via* two independent mechanisms.

## Introduction

A paradigm shift in recent years has given rise to the field of Systems/Network Pharmacology whose focus is identifying drug candidates that act *via* modulation of multiple networked targets.^1^,^2^ This focus is predicated on the thought that drugs possessing target promiscuity may result in enhanced efficacy. It is leading to a rethinking of the “magic bullet” approach involving drugs that bind and interact preferentially with a single disease target.^3^,^4^ This latter approach, while time-honored, is characterized by high drug attrition rates in clinical trials.^5^,^6^

**Fig. 1:**
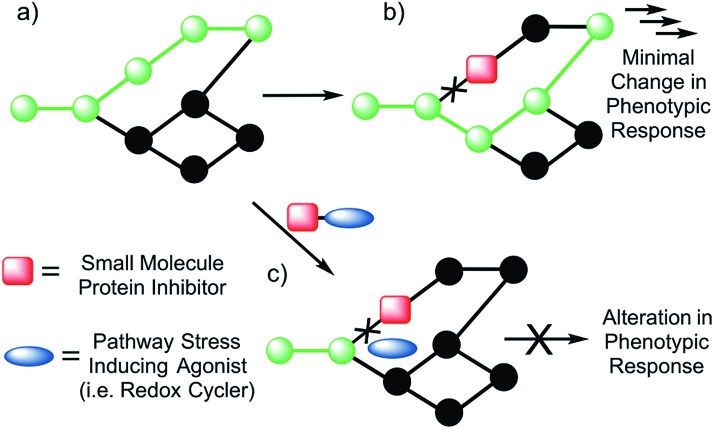
Schematic view of bimodal network targeting. (a) Normal (green) signal transduction within a generic biological network. (b) In the presence of a small molecule protein inhibitor, the pathway is shut down (black); however, no change in response is observed due to redirection of the signal transduction. (c) It is hypothesized that biochemical targeting with the same protein inhibitor in conjunction with a small molecule capable of inducing general pathway stress (*e.g.*, a redox cycler) will shut down the network, resulting in a greater alteration in the phenotypic response.

The emerging appeal of systems-based therapeutic approaches has prompted efforts to identify viable targets within biological networks (Fig. 1). Unfortunately, to date, random deletions or inhibition of specific proteins have typically led to poor phenotypic outputs due to the scale-free nature of biological networks.^7^ As a consequence, the targeting of single proteins or nodes within a biological system often does not lead to viable drug candidates (Fig. 1b). On the other hand, dual knockout yeast model studies have lent support to the suggestion that the simultaneous deletion of two genes can result in a phenotypic alteration under conditions where the targeting of a single gene will not.^8^,^9^ Developing a small molecule dual targeting approach to regulating and maintaining cellular networks is a current challenge with few known examples.^10^–^12^

**Fig. 2:**
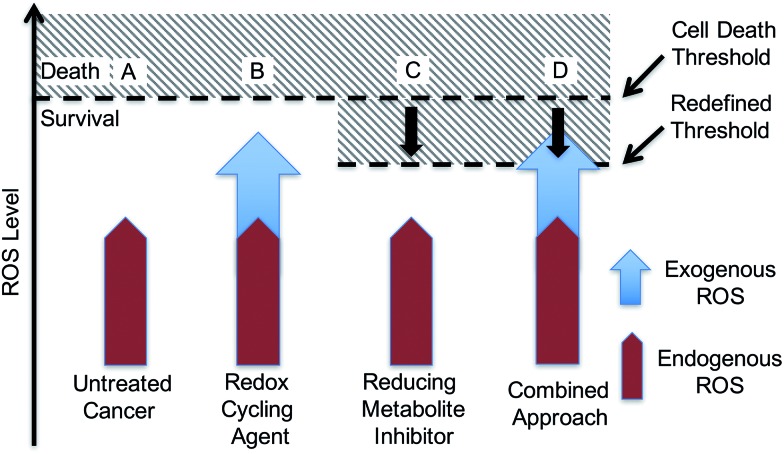
Mechanism based rationale regarding a dual targeting approach in drug design. (A) Elevated endogenous ROS that is tolerated by cancer cells. (B) Accentuating exogenous ROS by a single mechanism may not reach the cell death threshold. (C) Antioxidant inhibitors reduce the concentrations of reducing metabolites, thus lowering the cell death threshold. (D) The dual targeting approach involves the use of a redox cycler to accentuate exogenous ROS in combination with a reducing metabolite inhibitor to lower the cell death threshold. This combination is expected to overwhelm the system and drive it towards death.

With such considerations in mind, we have developed a new approach that involves the dual targeting of antioxidant response mechanisms. We believe that oxidative damage and endogenous prevention provide an ideal model for dual network targeting since (1) the antioxidant response pathway is overexpressed in several cancers, (2) effective targeting leads to alterations of growth phenotypes, and (3) normal cells are believed to have a greater capacity for reactive oxygen species (ROS) adaptation.^13^–^17^ Targeting the antioxidant network is a recognized strategy for anticancer development; however, there are limited examples of complexes that can pleiotropically modulate distinct mechanisms simultaneously.^18^–^21^ To achieve a systems-based approach to targeting the antioxidant pathway, we suggest that it would be beneficial to develop an agent that both reduces ROS tolerance (by inhibiting reducing metabolites) while increasing ROS production (Fig. 2). This would lead to antioxidant homeostasis being perturbed from both ends, thus overwhelming the network and promoting cell death (Fig. 1c and 2). Here, we present the results of a first study along these lines. Specifically, we present the synthesis, *in vitro*, and preliminary *in vivo* testing of a series of redox active, quinone-annulated gold(i) N-heterocyclic carbene complexes that both promote singlet oxygen generation and inhibit thioredoxin reductase (TrxR).

Thioredoxin reductase (TrxR) is a selenoenzyme that plays a central role within the antioxidant system. It regenerates thioredoxin (Trx) through an NADPH-dependent reduction of the active site disulfide bond (Cys32 & Cys35) present in oxidized Trx.^22^,^23^ The reduced form of Trx reacts with ROS and thus helps overcome oxidative stress. This has made inhibition of Trx/TrxR an attractive strategy for patients undergoing radiation therapy.^24^ Consistent with other types of cancer, TrxR is overexpressed in human lung carcinoma models (*e.g.*, the A549 cell line), providing a relevant model for antioxidant network targeting.^25^,^26^ Specific knockdown of TrxR by 90% (*via* siRNA), however, provided little to no phenotypic change in cell proliferation.^26^ In addition, treatment with auranofin, a Au(i) complex that targets TrxR, resulted in no difference in cell proliferation between TrxR knockdown A549 and A549 cells treated with mock siRNA. This robustness of TrxR is consistent with a highly networked endogenous antioxidant system that would require multiple modes of drug targeting to be suppressed in a therapeutically useful manner (Fig. 1b and c).

The overexpression and robustness of TrxR reported in several cancer models makes it a unique challenge within the context of network pharmacological drug development. Specific small molecule inhibition of TrxR yielded non-significant changes in cell growth, suggesting that a combined system approach is necessary to bypass the inherent redundancy.^26^ To explore this possibility we sought to develop a single molecular entity capable of both TrxR inhibition and redox cycling. In principle, this would both allow an increase in ROS production (through redox cycling) and a reduced ability to decrease the effects of ROS-based oxidative stress (through TrxR inhibition).

Quinones are venerable redox cycling agents. Under biological conditions, many quinones can accentuate ROS production beyond the buffering capacity of the cell. This is a feature that has long been appreciated in the context of cancer therapy,^27^–^30^ and one that is potentially useful in targeting the antioxidant pathway. Separate seminal work by Berners-Price and Filipovska led to an appreciation that appropriately designed gold(i) N*-*heterocyclic carbene (NHC) complexes can inhibit TrxR. This inhibition results from binding to the selenylsulfide/selenothiol redox center at the active site of TrxR.^31^–^34^ This has encouraged us and others to explore the utility of mono-NHC and bis-NHC gold(i) complexes as potential anticancer therapeutic agents with recent examples being efficacious in mammalian xenograft bearing models.^10^,^35^–^57^ We now suggest that using a quinone-bearing Au–NHC complex will allow a two-fold interruption of the antioxidant pathway *via* both overproduction of ROS and a decrease in TrxR-based ROS mediation.

**Fig. 3:**
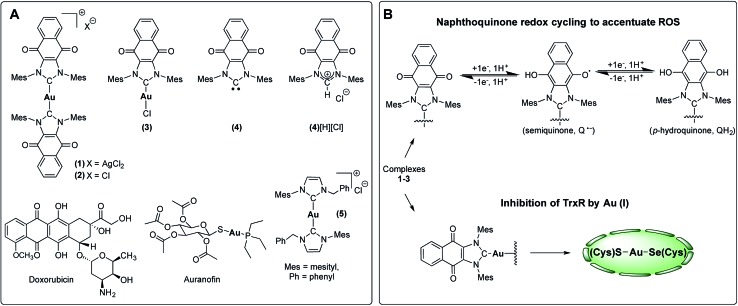
(A) Complexes studied and (B) proposed dual mechanism of action for naphthoquinone functionalized gold(i) complexes [NHC–Au–NHC][X] (X = Cl^–^ or AgCl_2_^–^, **1–3**).

To test this hypothesis, we have designed and synthesized the NHC gold(i) complexes **1–3** from the chloride anion salt of 1,3-dimesitylnaphthoquinimidazolium (**4**[H][Cl] Fig. 3c)^58^ and have assessed their potential for bimodal pathway targeting. Doxorubicin, an FDA-approved drug, auranofin, and the bis(1-benzyl-3-mesityl-imidazol-2-ylidene)-gold(i) chloride complex (**5**) were used as control complexes. Doxorubicin possesses a conjugated multi-ring quinone-based moiety capable of either DNA intercalation or ROS accentuation (depending on locus of action), while auranofin contains an Au(i)-phosphine coordination motif known to inhibit TrxR activity.^26^,^59^–^63^ Complex **5** contains no redox cycling component, but has been previously reported by us to inhibit TrxR.^10^

## Results and discussion

### Synthesis & characterization

**Scheme 1:**
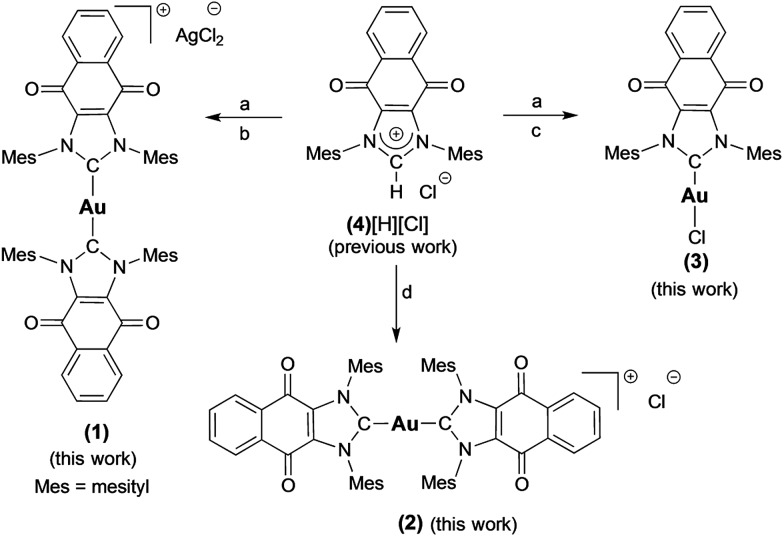
Preparation of complexes **1–3** from **4**[H][Cl]. a = Ag_2_O, CH_2_Cl_2_, b = 0.45 (C_4_H_8_S)AuCl, THF, c = (C_4_H_8_S)AuCl, THF, d = NaN(SiMe_3_)_2_, toluene, and 0.45 (C_4_H_8_S)AuCl.

Complex **1** ([(**4**)_2_Au][AgCl_2_]) was prepared in 82% yield by treating 1 equiv. of (**4**)Ag–Cl^58^,^64^ with 0.45 equiv. of (C_4_H_8_S)Au–Cl (Scheme 1).^65^ Proton NMR spectral analyses of **1** in CD_2_Cl_2_ proved consistent with the molecular structure of [(**4**)_2_Au]^+^. For instance, mesityl-CH_3_ hydrogen signals (*ortho*-CH_3_), corresponding to 24 hydrogen atoms, were observed at 1.63 ppm, while mesityl-CH_3_ hydrogen signals (*para*-CH_3_), corresponding to 12 hydrogen atoms, were observed at 2.43 ppm. A significant upfield shift (1.63 ppm) in the mesityl-CH_3_ hydrogen signals (*ortho*-CH_3_) was seen, and was taken as evidence for the presence of a [(**4**)_2_Au]^+^ subunit, as observed for other reported [bis(NHC)Au]^+^ complexes (wherein the corresponding signal resonates at 1.68 ppm in CDCl_3_).^66^ In the ^13^C NMR spectrum (CD_2_Cl_2_), a diagnostic chemical shift corresponding to C_carbene_–Au–C_carbene_ for **1** was observed at *δ*^13^C (Au–C_carbene_) = 192.6 ppm. This corresponds to a downfield shift compared to other reported [(NHC)–Au–(NHC)]^+^ complexes, such as bis(1-(ferrocenylmethyl)-3-mesitylimidazol-2-ylidene)-gold(i) (*δ*^13^C (Au–C_carbene_) = 183.2 ppm, CDCl_3_),^10^ bis(1,3-dimesitylimidazol-2-ylidene)-gold(i) tetrafluoroborate (*δ*^13^C (Au–C_carbene_) = 185.1 ppm, CDCl_3_),^66^ bis(1,3-dimethylimidazol-2-ylidene)-gold(i) bromide (*δ*^13^C (Au–C_carbene_) = 183.3 ppm, (CD_3_)_2_SO),^37^ and bis(1,3-dicyclohexylimidazol-2-ylidene)-gold(i) chloride (*δ*^13^C (Au–C_carbene_) = 180.4 ppm, (CD_3_)_2_SO)^37^ and is ascribed to the presence of the fused electron-withdrawing quinone that supports π-backbonding.^58^

An analogue of **1** ([(**4**)_2_Au][AgCl_2_]) containing a [Cl]^–^ counterion (*i.e.*, complex **2**) was also prepared. Complex **2** was synthesized in 75% yield by treating the free carbene **4** (1,3-dimesitylnaphthoquinimidazol-2-ylidene), generated *in situ,* with 0.45 equiv. of (C_4_H_8_S)Au–Cl (Scheme 1). As true for **1**, ^1^H NMR spectral analyses of **2** in CD_2_Cl_2_ proved consistent with the presence of the [(**4**)_2_Au]^+^ cation core. Using a modified literature procedure, a charge neutral mono-NHC functionalized gold(i) NHC (with NHC = 1-benzyl-3-mesityl-imidazol-2-ylidene) complex analogous to **1** (*i.e.*, **3**) was also prepared.^67^ It was obtained in 68% yield by treating 1 equiv. of (**4**)Ag–Cl with 1 equiv. of (C_4_H_8_S)Au–Cl. ^1^H NMR spectral analysis (CD_2_Cl_2_) of **3** was consistent with the proposed structure, whereas the ^13^C NMR (CD_2_Cl_2_) spectrum revealed that the diagnostic *δ* Au–C_carbene_ resonance appeared at 183.4 ppm. Again, this value is shifted downfield relative to other reported (NHC)Au–Cl complexes, for which corresponding resonances at *ca.* 168 ppm are seen.^68^ Gold complexes **1–3** were also characterized by ultraviolet-visible spectroscopy and infrared spectroscopy (see ESI†).

**Fig. 4:**
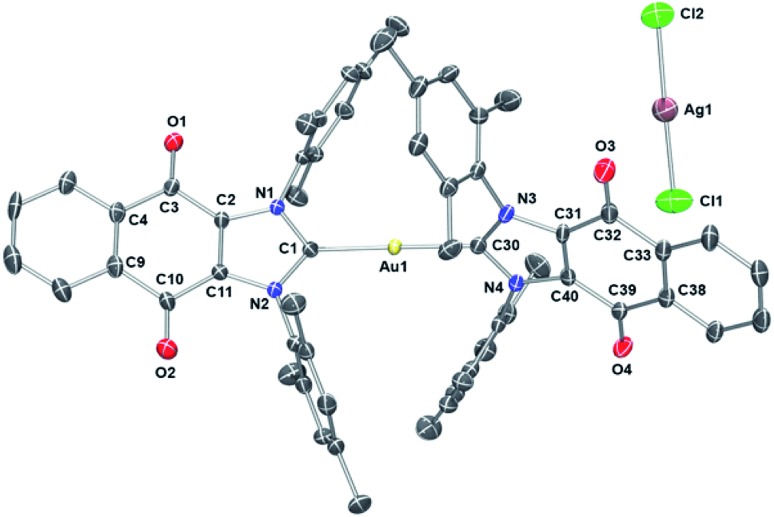
ORTEP plot of Compound **1** drawn using POV-Ray. Thermal ellipsoid plots are drawn at the 50% probability level and hydrogen atoms are omitted for clarity. Selected bond lengths (Å) and angles (deg): C1–N1, 1.347(6); C1–N2, 1.359(6); C30–N3, 1.356(6); C30–N4, 1.360(6); C1–Au1, 2.012(5); C30–Au1, 2.009(5); C3–O1, 1.217(6); C10–O2, 1.219(6); C39–O4, 1.207(6); C32–O3, 1.221(6); C11–C2, 1.350(7); C40–C31, 1.365(7); Cl1–Ag1, 2.3210(19); Cl2–Ag1, 2.3325(17); N1–C1–N2, 106.4(4); N3–C30–N4, 105.9(4); C1–Au1–C30, 172.87(19); Cl1–Ag1–Cl2, 178.46(8).

To assign the molecular structure unambiguously, X-ray diffraction quality single crystals of **1** and **3** were grown by slowly diffusing hexanes into a concentrated 1,2-dichloroethane solution (see ESI† for structure of **3**). Thermal ellipsoid plot of the resulting structure is presented in Fig. 4. In the case of **1**, a trans geometry was seen for the core [(**4**)_2_Au]^+^ cation with a C–Au–C bond angle of 172.8(2)° being observed. The Au–C_carbene_ bond distances of 2.012(5) Å and 2.009(4) Å are in agreement with those for other reported NHC–Au–NHC complexes.^52^,^63^,^66^,^68^,^69^ As inferred from the molecular structure of **1** (Fig. 4), the two carbene units are rotated around the gold atom with a torsion angle of 62.6(3)°. Presumably this twisting minimizes steric crowding.

### Electrochemistry

A series of electrochemical analyses, including cyclic voltammetry (CV) and differential pulse voltammetry (DPV), were carried out with [N*n*Bu_4_][PF_6_] in anhydrous dimethyl sulfoxide (DMSO) in order to evaluate electronic properties of compounds **1–3** and **4**[H][Cl]. Key half-wave reduction potentials for **1–3** and **4**[H][Cl], obtained from DPV measurements, are summarized in Table 1. In the CV measurements (scan rate = 100 mV s^–1^), all four compounds (**1–3** and **4**[H][Cl]) displayed cathodic waves that occur in two sequential steps in which the first wave is completely reversible and the second wave is quasireversible at a 0.1 mV s^–1^ scan rate; these are labeled as a and b in Table 1.^70^,^71^ These electrochemical features were attributed to the reduction of the quinone moiety to first produce the semiquinone radical (NQ^–^) and then produce the quinone dianion (NQ^2–^) forms of compounds **1–3** and **4**[H][Cl].^70^,^71^ The quinone reduction potential in **4**[H][Cl] occurs at –0.38 V, the lowest of all the molecules studied, indicative of a positively charged imidazolium ring. The quinone couple at –0.42 V observed for compound **1** is ascribed to the presence of bis(NHC). The same wave in compound **3** appears at a more negative potential (–0.47 V), presumably due to greater trans effects excreted by quinone annulated NHC ligand than the metal-bound chloride. This analysis agrees well with the differences in the observed *δ*^13^C (Au–C_carbene_) resonances for compounds **1** and **3**.

**Table 1:** Electrochemical analysis of compounds **1–3** and **4**[H][Cl]. The potentials were obtained from differential pulse voltammetry measurements in DMSO using 0.1 M [N(*n*Bu)_4_]^+^[PF_6_]^–^ as the supporting electrolyte, 0.1 mM analyte, and referenced *vs.* SCE. See the ESI for the corresponding cyclic voltammograms and differential pulse voltammograms

Compound	*E*_1/2_ ^*a*^ (V) DPV	*E*_1/2_ ^*b*^ (V) DPV
Compound **1**	–0.42	–1.31
Compound **2**	–0.46	–1.31
Compound **3**	–0.47	–1.31
Compound **4**[H][Cl]	–0.38	–1.15

^*a*^Assigned as the first reduction, formation of semiquinone radical (NQ˙^–^).

^*b*^Assigned as the second reduction, formation of quinone dianion (NQ^2–^).

Having studied the electronic properties, we sought to probe the stability and electronic nature of **1** upon reduction by means of UV-vis spectroelectrochemistry. Upon bulk electrolysis of compound **2** at a potential of –1.5 V using a special electrochemical cell, reduced quinone species were generated and simultaneously probed using UV-vis spectroscopy. Characteristic absorbance features ascribable to reduced quinone moieties were observed.^64^ The original UV-Vis spectral trace of compound **2** can be obtained after reduction (NQ → NQ^2–^) followed by subsequent oxidation (NQ^2–^ → NQ) (see ESI†). These findings provide support for the reduced species being stable under the conditions of electrochemical analysis.

### Cell proliferation assays

**Fig. 5:**
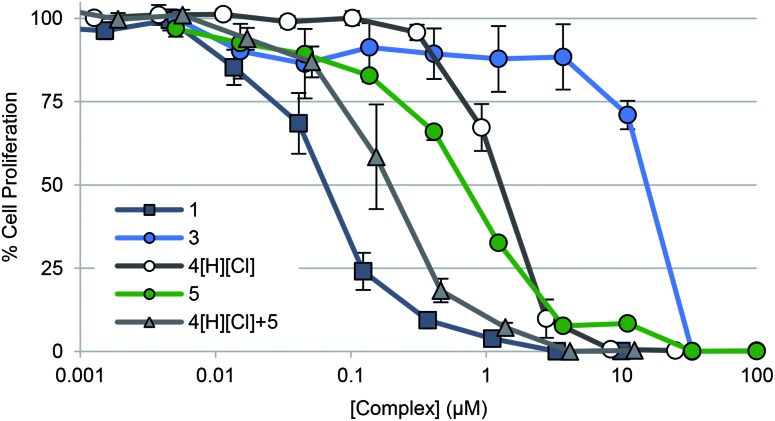
Cell proliferation profiles of A549 lung cancer cells treated with **1**, **3**, **4**[H][Cl], **5**, and a 2 : 1 molar concentration of **4**[H][Cl] and **5** (cocktail), respectively. Data for doxorubicin and auranofin are not shown for clarity purposes but are provided in the ESI† as well as in Table 2. Error bars represent the standard error of the mean.

To gauge the ability of each complex to inhibit cancer cell growth, A549 lung cancer cells were treated with **1–3**, **4**[H][Cl], doxorubicin, and auranofin in a dose responsive manner. Cellular vitality (*i.e.*, mitochondrial reductase activity) was then quantified colorimetrically post treatment using 3-(4,5-dimethylthiazol-2-yl)-2,5-diphenyltetrazolium bromide (MTT assay) (Table 2, Fig. 5). Dose responsive treatment of A549 cells with doxorubicin and auranofin provided growth inhibition curves and IC_50_ values of 0.103 ± 0.023 μM and 1.67 ± 0.05 μM, respectively. These values were similar to those previously reported.^10^,^61^ In the case of the gold(i) NHC quinone complex **1**, the corresponding IC_50_ value was determined to be 0.073 ± 0.016 μM. A similar value was recorded in the case of complex **2** (see ESI†). Complex **3** was essentially inactive (*i.e.*, >150× less potent than 1).

**Table 2:** Cell proliferation data in A549 lung cancer cells

Compound	IC_50_ (μM)	Std error (+/–)	Fold difference relative to 1
Doxorubicin	0.103	0.023	1.41
Auranofin	1.67	0.05	22.9
**1** ^*a*^	0.073	0.016	1
**2**	0.075	0.013	1
**3**	12.06	0.18	165
**4**	0.994	0.12	13.6
**5**	0.71	0.06	9.72
**4**[H][Cl] + **5**^*b*^	0.197	0.057	2.70

^*a*^Students *t*-test (unpaired) provided a *p*-value <0.05 when **1** was compared to **3**, **4**[H][Cl], **5**, and cocktail (**4**[H][Cl] + **5**).

^*b*^Cocktail dosing entailed a 2 : 1 molar ratio of **4**[H][Cl] and **5**, respectively. This dosing reflects the relative component stoichiometry in complex **1**.

To determine the relative contribution of the individual components present in **1** (*i.e.*, the quinone moiety *vs.* the Au(i)–NHC subunit), positively charged complexes containing a naphthoquinone (*i.e.*, **4**[H][Cl]) and the [(NHC)_2_Au]^+^ core (*i.e.*, **5**) were also studied; they gave IC_50_ values of 0.99 ± 0.12 μM and 0.71 ± 0.06 μM, respectively. Improved antiproliferative activity (IC_50_ = 0.197 ± 0.057 μM) was observed when A549 cells were exposed to a combination of **4**[H][Cl] and **5** in a 2 : 1 molar ratio that matches their stoichiometric ratio in 1. However, this combination was not as effective as complex **1** (by a factor of 2.7).

Further anti-proliferation studies were carried out with complex **1** and its naphthoquinone component **4**[H][Cl] using the following cell lines: A2780 ovarian (a wt-p53 cell line sensitive to platinum treatment), 2780CP ovarian (isogenic to A2780 but expressing multi-drug resistance (MDR)), and PC-3 prostate (p53 null) (Table 3). While both complexes reduced proliferation in all three cell lines, complex **1** was found to be statistically more potent in each cell line relative to **4**[H][Cl].

**Table 3:** IC_50_ values of the naphthoquinone Au(i)–NHC complex **1** and the naphthoquinone imidazolium salt **4**[H][Cl] in various cancer cell lines^*a*^

Compound	A549 lung	A2780 ovarian	2780CP ovarian	PC-3 prostate
**1** ^*b*^ ^,^ ^*c*^	0.073 ± 0.016	0.026 ± 0.007	0.054 ± 0.006	0.096 ± 0.017
**4**[H][Cl]^*d*^	0.994 ± 0.120	0.159 ± 0.058	0.626 ± 0.117	0.136 ± 0.020

^*a*^Error is represented as standard error from the mean.

^*b*^Students *t*-test (unpaired) revealed **1** was significantly more potent than **4**[H][Cl] in every cell line (*p*-value <0.005 for A549, A2780, 2780CP; *p*-value <0.05 for PC-3).

^*c*^Students *t*-test (unpaired) revealed that the potency was different in A2780.

^*d*^Students *t*-test (unpaired) revealed no difference in potency between the A2780 and PC3 cell lines. 2780CP was significantly different from A549, A2780, and PC-3 (*p*-value <0.0005).

### Cellular uptake and interaction with serum proteins

**Fig. 6:**
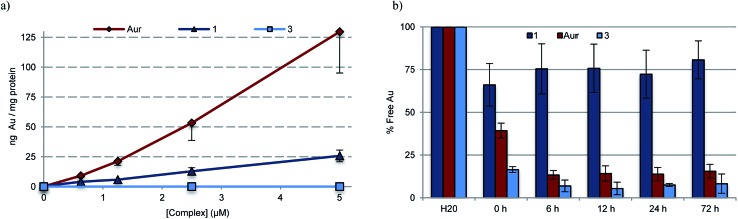
(a) ICP-MS detection of intracellular Au levels as an indicator of complex uptake into A549 lung cancer cells. Students *t*-test (unpaired) of **1** (2.5 μM) compared to auranofin (2.5 μM) provided *p*-value <0.05, indicating statistical significance. A comparison of **1** to **3** (*p*-value >0.2) revealed no statistical significance. (b) Percent of free Au (non-protein bound) within samples of fetal bovine serum treated with 25 μM **1**, **3**, and auranofin. Error bars represent the standard error from the mean.

To quantify the extent to which variations in cellular uptake might account for the differences in anti-proliferative efficacy seen for the various gold(i) complexes of this study, inductively coupled plasma mass spectrometry (ICP-MS) was used to detect intracellular Au levels (Fig. 6a). In brief, cell cultures of A549 were treated with varying doses of **1**, **3**, **5**, and auranofin, collected and digested, and quantitatively assessed for intracellular Au content. It was found that regardless of dose, a 2–5 fold increase in intracellular Au concentrations was seen in samples treated with auranofin as compared to complex **1**. In the case of **3**, a neutral complex, no intracellular Au was detected under conditions identical to those used to test complex **1**. The intracellular Au levels were found to be identical in the case of complexes **1** and **5** (see ESI†).

To assess potential drug protein interactions, samples of fetal bovine serum (FBS) were treated with 25 μM **1**, **3**, and auranofin prior to incubating at 37 °C. Aliquots were taken and the free Au (non-protein bound, methanolic extracts) content was analyzed by ICP-MS (Fig. 6b). As expected, the free Au content in the FBS samples treated with auranofin decreased in a time dependent manner.^72^,^73^ A similar reduction in free Au was observed for FBS samples treated with complex **3**. In contrast, minimal changes in the free Au levels were seen as a function of time in the samples containing complex **1**. This result is consistent with the notion that Au(i)–NHC 1 enters the cell *via* different mechanism than auranofin (Fig. 6a). In addition, the protein binding differences between **1** and **3** could explain the relatively reduced potency seen in the case of **3**.

### Accentuation of reactive oxygen species (ROS)

**Fig. 7:**
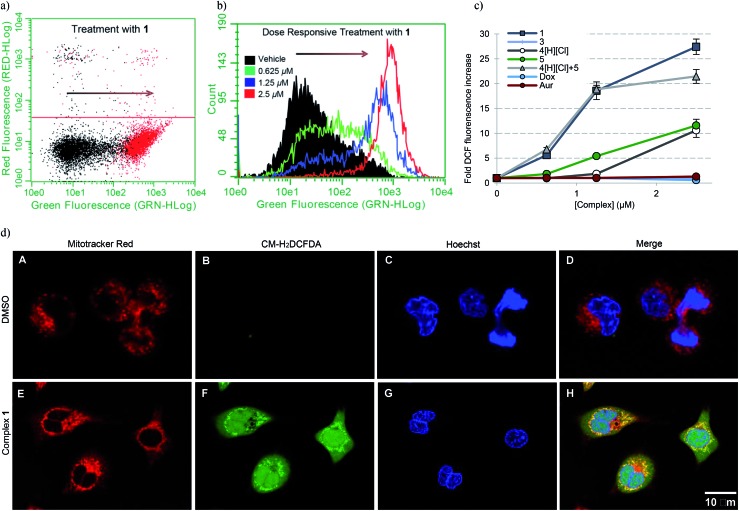
(a) A549 cell population shift (black = vehicle treatment, red = treatment with 2.5 μM **1**) indicating accentuation of intracellular ROS *via* flow cytometry. (b) Dose responsive cell population shift of A549 cells treated with **1**. (c) Dose responsive accentuation of intracellular ROS with various complexes post 6 h incubation. Error bars represent the standard error of the mean. Treatment of A549 cells with 100 μM H_2_O_2_ was used as a positive control (see ESI†). Students *t*-test (unpaired) of **1** compared to the cocktail provided *p*-values >0.05 suggesting no statistical significance. Comparison of **1** to either **4**[H][Cl] or **5** individually (*p*-values < 0.005) revealed a statistically significant difference in both cases. (d) Confocal microscopy studies illustrating mitochondria specific ROS generation in A549 cells treated with 1.25 μM complex **1**.

To establish whether or not the complexes of this study would increase intracellular ROS levels, A549 cells were treated with each complex in a dose responsive manner. ROS fluctuations were monitored post treatment *via* flow cytometry using the fluorescein-based general ROS indicator (5-and-6)-chloromethyl-2′,7′-dichlorodihydro-fuorescein diacetate, acetyl ester (CM-H_2_DCFDA). Following treatment with 2.5 μM **1**, a 27-fold fluorescence associated cell population shift was observed (Fig. 7a), a finding taken as indicative of a significant increase in intracellular ROS in the case of this complex. A dose dependence was also seen (Fig. 7b). Upon treatment with the individual components of **1** (*i.e.***4**[H][Cl] and **5**), a more modest increase in ROS was observed (∼11-fold increase at the 2.5 μM dose level in each case), while minimal or no ROS increase was observed in the case of **3**, auranofin, or doxorubicin (Fig. 7c). When A549 cells were exposed to a 2 : 1 molar ratio of **4**[H][Cl] and **5** a dose-dependent increase in ROS was observed that statistically similar to that produced by **1**. This is rationalized in terms of the ROS enhancement produced upon exposure to the individual components present in **1** being additive and not synergistic.

To further elucidate the subcellular loci of ROS accentuation, confocal microscopy was employed to fluorescently image A549 cancer cells treated with vehicle (DMSO) and 1.25 μM complex **1** (Fig. 7d). All cells were selectively stained for visualization of ROS accentuation (green, CM-H_2_DCFDA), mitochondria (red, Mitotracker Red), and nuclei (blue, Hoechst). No ROS accentuation was observed in cells treated with DMSO. A549 cells treated with complex **1** resulted in a general green fluorescence increase with localized areas of higher green fluorescence (Fig. 7d, image F). Once merged, evident overlap of localized ROS accentuation with mitochondria (red) suggests that ROS accentuation is arising from mitochondria (Fig. 7d, image H).

### Inhibition of thioredoxin reductase

**Fig. 8:**
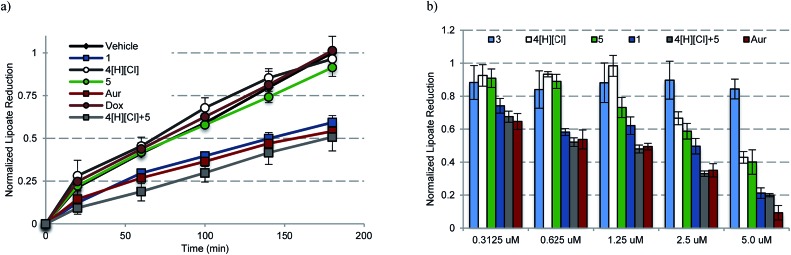
(a) Time dependent inhibition of TrxR activity in A549 cells treated with 0.6125 μM of the indicated compound for 180 min. Complex **1** was statistically different than vehicle, **4**[H][Cl], **5**, and Dox (*p* values < 0.005), and similar to auranofin and **4**[H][Cl] + **5** (*p* values > 0.1) by the (unpaired) Students *t*-test. (b) Dose responsive inhibition of TrxR activity in A549 cells treated with 0.156–5.0 μM compound for 6 h and then incubated for 3 h with lipoate. Doxorubicin data are not shown for clarity (see ESI†).

To assess whether any or all of the present gold complexes could serve as TrxR inhibitors, standard tests involving the reduction of the oxidized form of the cell-permeable cofactor lipoate to its corresponding reduced form, dihydrolipoate, were carried out. Briefly, plateau phase A549 cells were exposed to variable doses of complexes **1**, **3**, **4**[H][Cl], a 2 : 1 molar ratio of **4**[H][Cl] and **5** (cocktail), auranofin, and doxorubicin for 6 h. Post treatment, the live cells were monitored colorimetrically over 180 min for their ability to reduce lipoate (Fig. 8a). Depending on the incubation concentration distinct differences in the time dependent inhibition of TrxR are evident. At low concentrations (0.1–0.6 μM), inhibition of TrxR was apparent in A549 cells exposed to **1**, auranofin, and **4**[H][Cl] + **5**, while little to no inhibition was seen in the case of **3–5** or doxorubicin. At higher concentrations (1.25–5.0 μM), inhibition of TrxR by **4**[H][Cl] and **5** became evident, while **3** or doxorubicin remained inactive over the full concentration range used in the study (see ESI†). We thus propose that complex **1** will be able to act as both a TrxR inhibitor and a general agonist of oxidative stress.

### Induction of apoptosis

**Fig. 9:**
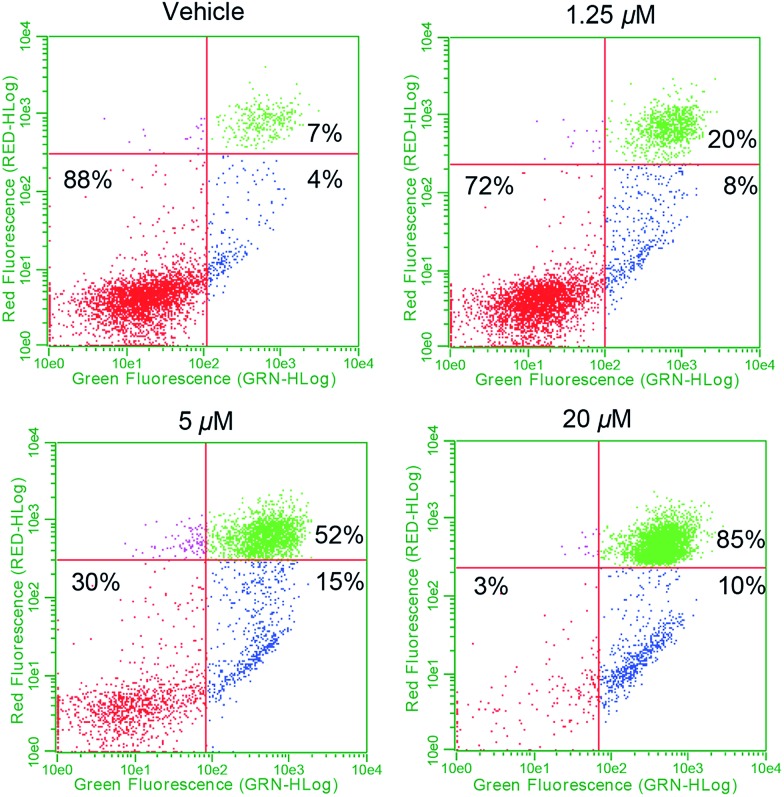
Cell death *via* apoptosis as detected using flow cytometry. Study is suggestive of the activation of apoptosis by **1** due to the presence of two separate annexin-V positive populations representing early stage (bottom right) and late stage (top right) apoptosis.

To determine whether complex **1** also promotes apoptosis, flow cytometry studies in conjunction with annexin-V staining were carried out. In brief, plated exponential growth phase A549 cells were exposed to various concentrations of **1** and incubated for 24 h. At that point, all cells (adhered and floating) were collected, washed, and stained with fluorescein-labeled annexin-V and propidium iodide (PI) and subjected to flow cytometry (Fig. 9). At low doses, evidence of early stage apoptosis was seen, as inferred from the binding of annexin-V to the still-intact and impermeable cell membrane (resulting in FITC-only fluorescence). As the dose escalation progressed, a larger percentage of late stage apoptosis/necrotic (FITC positive and PI positive from staining of nuclear material) cells became evident. Treatment of A549 cells with doxorubicin (a known inducer of apoptosis) provided similar results in both the early and late stage apoptotic quadrants (see ESI†). On this basis, we conclude that complex **1** induces controlled cell death *via* an apoptotic mechanism.^61^

### Toxicity and efficacy studies in zebrafish

**Fig. 10:**
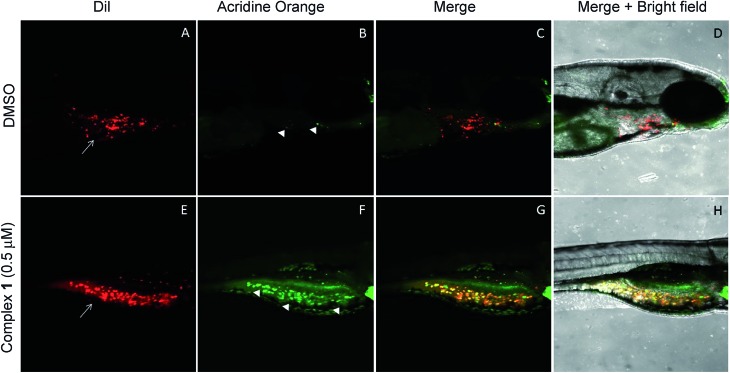
Complex **1** induces tumor specific cell death in Zebrafish tumor xenografts. A–D are lateral view of 3 day old zebrafish tumor xenografts treated with 0.5 μM DMSO and E–H are lateral views of 3 day old zebrafish tumor xenografts treated with 0.5 μM complex **1**. A and E shows the DiI labeled (Red, white arrow) A549 lung cancer cells in the DMSO and complex **1** treated xenografts, respectively. B and F shows Acridine Orange labeled (Green, white arrowheads) dead A549 cells within the DMSO and complex **1** xenografts, respectively. Whereas DMSO treated xenografts display very few dead cells (B), complex **1** treated xenografts display cell death of majority of tumor cells (F). C and G are the merge of DiI and Acridine orange staining of xenografts where yellow/orange indicate dead cells. D and H are the bright field images of DMSO and complex **1** treated xenografts showing no non-specific cell death in the developing zebrafish larvae.

The anticancer activity of the complex **1** was tested using a qualitative high throughput zebrafish tumor xenograft model (IACUC # I13009).^74^ First, zebrafish embryos were divided into 7 groups at an average of 65 embryos per group. Each group was treated with vehicle (DMSO) or complex **1** at variable concentrations to identify the maximum tolerable dose (MTD) (Fig. S29†). A dosing of 0.5 μM was found to induce no observable toxic effect relative to vehicle (*p*-value > 0.1) and was deemed to be the MTD for zebrafish embryos. Therefore, efficacy studies, using zebrafish bearing human tumor xenografts were carried out with complex **1** being administered at the 0.5 μM concentration level.^75^–^77^ Briefly, live human lung cancer cells (A549) were labeled with CM-DiI (red) and only live cells were transplanted *via* injection into the perivitelline space of 30 zebrafish embryos 24 hours post fertilization (hpf).^74^,^78^,^79^ Tumor inoculated zebrafish embryos were allowed to grow for one day till 48 hpf. This allows for establishment of the cancer cells in the host zebrafish embryos. At 48 hpf, the xenograft bearing zebrafish embryos were split into 2 groups (15 embryos per group) and treated with vehicle (DMSO) or complex **1** at 0.5 μM for one additional day (72 hpf), and cancer cell death was observed using acridine orange staining (green). Live zebrafish-A549 tumor xenografts treated with DMSO display features consistent with the presence of tumor cells (red, white arrows in Fig. 10A). On the other hand, few, if any, tumors and little evidence of host cell apoptosis was seen with acridine orange staining (green, arrowhead in Fig. 10B–D). Finally, live zebrafish-A549 tumor xenografts (red, white arrows in Fig. 10D) treated with complex **1** showed evidence of apoptosis for the majority of tumor cells under conditions of acridine orange staining (green, arrowhead in Fig. 10E and yellow or orange cells in Fig. 10F and G).

## Discussion

Naphthoquinone functionalized N-heterocyclic carbene supported gold(i) complexes (**1–3**) were designed to test whether the inhibition of TrxR in parallel with an increase of network stress (higher levels of ROS) would lead to an enhanced phenotypic response (reduction in cell growth). The Au(i)–NHC and naphthoquinone moieties of the present study were specifically chosen to (1) inhibit TrxR *via* irreversible binding of an Au(i) center to the selenothiol-containing active site and (2) accentuate ROS *via* redox cycling of the naphthoquinone moieties. The goal was to achieve these complementary functions using a single molecule. The use of a single molecule that achieves two targeting functions concurrently is expected to allow for better control ultimately over such key design features as metabolism, uptake, localization, and clearance, to name a few. With such considerations in mind, two bis-carbene Au complexes with different counter anions were prepared (*i.e.*, **1** and **2**). While **2** contains a biologically compatible counterion [Cl^–^], **1** contains an [AgCl_2_]^–^ which is often an artifact from the transmetalation route to [(NHC)–M–(NHC)]^+^ complexes (M = Au or Ag) and has been previously reported by some to be of biological influence.^80^–^84^ Initial side-by-side comparisons revealed no appreciable difference between **1** and **2** in their ability to inhibit cell proliferation, induce exogenous ROS, or inhibit TrxR activity. ICP-MS analysis of cells treated with **1** and **2** showed similar Au uptake between complexes. Considering the intracellular uptake of the [AgCl_2_]^–^ counterion of **1**, a 7 : 1 Au : Ag uptake ratio was observed *via* ICP-MS. This suggests that the [AgCl_2_]^–^ minimally enters the cell possibly due to ion exchange with salts within the cell culture medium (see ESI† for a complete comparison between **1** and **2**). Detailed studies were thus carried out with **1** and various controls. As noted in the Results section above, in cell proliferation studies, this complex proved much more active than auranofin (23-fold), **4**[H][Cl] (∼14-fold), **5** (∼10-fold), or a 2 : 1 mixture of the latter species (Fig. 5 and Table 2). The stark (*i.e.*, ≥10×) increase in potency seen for **1** relative to its individual parts (*i.e.*, **4**[H][Cl] and **5**) leads us to suggest that both the Au(i) NHC and quinone moieties contribute to the observed antiproliferative activity. Furthermore, the ability of **1** to inhibit cell proliferation was found to be 165-fold greater than the monoNHC-Au(i) complex **3**.

ICP-MS data provide support for the conclusion that complexes **1** and **5** enter cells more effectively than **3** (Fig. 6 and S20†). Drug uptake is multifactorial, and the varying cellular uptake levels seen for the various Au(i) complexes could reflect differences in lipophilicity, the presence of positive charges facilitating passive diffusion, and complex-serum protein interactions.^32^ Auranofin is known to bind serum proteins, such as human serum albumin and bovine serum albumin, which are thought to provide a transport mechanism into the cell.^72^,^73^,^85^,^86^ On the other hand, the reduced uptake seen for **3** (in contrast to **1** and **5**) is ascribed to irreversible sequestration by serum proteins, a conclusion that is consistent with recent structural work showing that mono-NHC ligated Au(i) complexes bind lysine residues within protein models.^87^,^88^ On the basis of the present work, we propose that such irreversible binding be avoided if the goal is to achieve cell uptake and targeting of the antioxidant network (Fig. 6a and b).^72^,^73^,^85^,^86^

Based on ICP-MS analysis, we conclude that complex **1** is less reactive towards serum proteins than auranofin. This corresponds to an increase in complex stability that we ascribe to the differences in Au-ligation (*i.e.*, NHC *versus* phosphine). Notwithstanding its increased stability relative to auranofin, complex **1** was found to inhibit well the activity of TrxR. This finding is ascribed to complex **1** undergoing facile exchange with Se containing biomolecules (*i.e.*, TrxR) with no appreciable reactivity towards other common biological nucleophiles, including protein thiols or amines. The combined benefits of a decrease in serum protein reactivity (which prevents loss of active Au) while retaining effective TrxR inhibition provide for a potential increase in therapeutic benefit and an enhanced safety window.^32^

A dose responsive increase in ROS was observed in A549 cells for several of the complexes, which culminated in a maximal 27-fold increase at 2.5 μM in the case of **1**. This ROS accentuation by **1** was found to be localized to the mitochondria as evidenced by confocal microscopy studies. In contrast to what was seen in the cell proliferation studies, 2 : 1 molar mixtures of **4**[H][Cl] and **5** (a “cocktail” mimicking the stoichiometry of the subunits within **1**) engendered statistically similar levels of ROS to that of complex **1**. The difference between the growth and ROS phenotypes is consistent with the notion that two different modes of action (ROS generation and TrxR inhibition) are responsible for the observed biological activity. In fact, complex **1**, in contrast to previous systems we have studied,^10^ was found to inhibit TrxR activity strongly (*i.e.*, at levels similar to auranofin (Fig. 8)). This was also true for the 2 : 1 stoichiometric mixture of **4**[H][Cl] + **5** (cocktail), but not for either of the components (**4**[H][Cl] or **5**) when tested individually. This leads us to suggest that the increased TrxR inhibition by **1** is due to the presence of the naphthoquinone moieties and not due to potential differences in Au-carbene metal ligand interactions. The statistical indifference between **1** and the 2 : 1 stoichiometric mixture of **4**[H][Cl] and **5** regarding ROS accentuation and TrxR inhibition is in stark contrast to the 2.7-fold difference in growth phenotypes. We ascribe the increase in biological potency relative to what one might expect based on a simple sum of the chemical and enzymatic inhibition effects provided by the individual components (*i.e.*, **4**[H][Cl] and **5**) to the effect of conjugation. The tethered system 1 helps assure the concurrent subcellular localization of both active species, namely the naphthoquinone and the NHC-complexed Au centers.

Doxorubicin, a conjugated anthracycline possessing a quinone moiety, is thought to mediate its anticancer effect through inhibition of topoisomerase II *via* DNA intercalation. However, it has been established that doxorubicin localization to healthy cardiac tissue induces cellular stress and dose limiting toxicity *via* mitochondrial ROS accentuation.^29^,^60^,^62^,^63^ This duality of action led us to question whether the naphthoquinone complexes of the present study (*e.g.*, **1** and **4**[H][Cl]) would also interact with DNA, mediating an effect apart from their targeted ROS producing function. To test this possibility, thermal denaturation studies with short DNA duplexes were carried out. Significant DNA stabilization was observed in the case of doxorubicin; however, no thermal stabilization of DNA by **1** or **4**[H][Cl] was observed under the study conditions (see ESI†). This is consistent with complex **1** and doxorubicin operating *via* different mechanisms. However, both induce cell death *via* apoptosis as inferred from the formation of two positive annexin-V populations (+PI, –PI) (Fig. 9).

Complex **1** was also found to inhibit cell proliferation across several cancer cell lines displaying varying p53 status and drug resistance profiles, namely PC3 prostate (p53 null), A2780 ovarian (wt-p53 platinum sensitive), and 2780CP (isogenic partner to A2780 displaying multidrug resistance (MDR)) (Table 3). The gold-free naphthoquinone **4**[H][Cl] was also tested. Across all cell lines, complex **1** proved more potent than **4**[H][Cl]. Of the four cell lines tested, it should be noted that complex **1** displayed higher potency in the A2780 cell line, as compared to the A549, 2780CP and PC-3 cell lines (where similar potency levels were observed). Regarding naphthoquinone **4**[H][Cl], a stronger antiproliferative effect was observed in the A2780 and PC-3 cell lines relative to 2780CP and A549. These trends may reflect differing pharmacological profiles that warrant further exploration.

To assess the utility of complex **1***in vivo*, a zebrafish xenograft tumor model was used.^74^ Such zebrafish models are attractive as they (a) provide a qualitative high throughput cancer drug screen in a system that maintains the complex physiology of the human tumor and (b) allow for the assessment of dose limiting toxicity through non tumor specific cell death. This efficacy/toxicity system presents a preliminary valuation for cancer death selectivity and therapeutic index. Using this kind of model, it was found that zebrafish embryos tolerated well a 0.5 μM dose of complex **1** with no significant host cell apoptosis. Moreover, at that dose, cancer specific cell death was seen in zebrafish embryos bearing human A549 lung cancer xenografts. Based on this finding, the induced apoptosis is thought to be largely, if not completely, localized within the tumor xenografts. These preliminary studies validate the ability of complex **1** to selectively induce cancer cell death *in vivo* at levels that do not produce toxic effects and warrant further investigation in mammalian murine models.

## Conclusions

Herein we report that targeting a highly networked antioxidant regulator (TrxR) results in a greater phenotypic alteration (cell proliferation) when combined with a network stress inducer (*i.e.*, accentuation of oxidative stress *via* redox cycling). The incorporation of redox cycling naphthoquinone subunits within an Au(i)–NHC core to give complexes such as **1** and **2** leads to an enhancement in the anti-proliferative activity. This enhancement is ascribed to the combination of ROS accentuation and TrxR inhibition provided by the individual components and to the fact that the species are tethered to one another, thus controlling co-localization (*e.g.*, uptake and clearance). The anticancer activity and low toxicity seen in the zebrafish-A549 tumor xenograft provides support for the notion that complex **1** warrants further study as a potential anticancer agent. We view these preliminary findings as promising considering clinical drugs such as anthracyclines and platinums induce organ specific toxicities that are dose limiting. More broadly, the success of **1** relative to various controls, including auranofin and doxorubicin, provides “proof-of-principle” support for the suggestion that targeting key cancer-related pathways *via* multiple modes of action may have utility as a therapeutic paradigm. Further tests of this hypothesis are ongoing in our laboratories.

## Author contributions

The manuscript was written through contributions of all authors. All authors have given approval to the final version of the manuscript.

## Abbreviations

TrxR: Thioredoxin reductase
ROS: Reactive oxygen species

## Supplementary Material

Supplementary information

Crystal structure data
