# Supplementary material for: Dual targeting of the cancer antioxidant network with 1,4-naphthoquinone fused Gold(i) N-heterocyclic carbene complexes
Source: Chem Sci. 2017 Jul 21;8(9):5918–29. doi: 10.1039/c7sc02153d (PMC5859730; doi:10.1039/c7sc02153d)
Supplement: Supplementary file 1 [file SC-008-C7SC02153D-s001.pdf]

Electronic Supplementary Material (ESI) for Chemical Science.
This journal is © The Royal Society of Chemistry 2017

Dual Targeting of the Cancer Antioxidant Network with Naphtho-quinone Fused Gold(I) N-heterocyclic Carbene Complexes

R. McCall,[a] M. Miles,[b] P. Lascuna,[c] B. Burney,[a] Z. Patel,[a] K. J. Sidoran,[d] V. Sittaramane,[c] J. Kocerha,[a] D. A. Grossie,[b] J. L. Sessler,[e] K. Arumugam,[b,]* and J. F. Arambula[a,e]*

[a] Department of Chemistry, Georgia Southern University, Statesboro, GA 30460, USA

[b] Department of Chemistry, Wright State University, Dayton, OH 45435, USA

[c] Department of Biology, Georgia Southern University, Statesboro, GA 30460, USA

[d] Department of Chemistry, St. Bonaventure University, St. Bonaventure, NY 14778, USA

[e] Department of Chemistry, University of Texas, Austin, TX 78712-1224, USA

Supporting Information

*E-mail: kuppuswamy.arumugam@wright.edu; jfarambula@cm.utesxas.edu

Table of Contents

Materials and Methods S2

Synthesis S2-S3

X-ray Crystallography S3-S5

Electrochemical Data S6-S9

^{1}H and ^{13}C NMR Spectra S10-S12

UV-Vis Absorption Spectra S13-S14

Biological Preparation and Assays S15-S17

Biological Data S18-S21

Comparison assays between Cl and $AgCl_2$ counterions S21-S22

Toxicity studies in Zebrafish S23

References S23

Materials and Methods

1,3-Dimesitylnapthoquinimidazolium chloride,[1-2] (1,3-dimesityl-4,5-naphthoquino-imidazol-2-ylidene)-silver(I) chloride,[1-2] (C_4H_2S)AuCl[3] were prepared according to the literature procedures. All other reagents were purchased from commercial sources and used as received, including: [((CH_3)$_3$Si)$_2$N]Na (NaHMDS) and silver(I) oxide. CD_2Cl_2 (99.9%) was purchased from Acros Laboratories in glass ampules and used as received. Solvents were either dried with a solvent purification system from the Inert Innovative Technology, Inc. (dichloromethane, diethyl ether, hexanes, tetrahydrofuran and toluene) or freshly distilled over 3 Å molecular sieves and degassed using three consecutive freeze-pump-thaw cycles prior to use. UV-vis spectra were obtained at ambient temperature with a Hewlett-Packard 8452A diode array spectrophotometer with molar absorptivities reported in M^{-1} cm^{-1}. ^1H and ^{13}C NMR spectra were recorded on Bruker 300 MHz spectrometer. Spectra were referenced to the residual solvent as an internal standard, for ^1H NMR: CD_2Cl_2, 5.32 ppm and ^{13}C NMR: CD_2Cl_2, 53.84 ppm. High-resolution mass spectra (HRMS) were obtained with an Agilent Technologies 6530 Accurate Mass Q-TOF LC/MS (ESI) and are reported as m/z (relative intensity). Electroanalytical measurements were performed on a CHI620E electrochemical workstation using a silver wire quasi-reference electrode, a platinum disk working electrode and a Pt wire auxiliary electrode in a three-electrode cell under an atmosphere of nitrogen. The electrochemical measurements were performed using 1.0 mM solutions of the analyte in dry DMSO with 0.1 M [N(*n*Bu)$_4$][PF_6] as the electrolyte and ferrocene (Fc) as the internal standard. Differential pulse voltammetry measurements were performed with 50 mV pulse amplitudes and 2 mV data intervals. All potentials listed herein were determined by differential pulse voltammetry and referenced to a saturated calomel electrode (SCE) by shifting ferrocene$^{0/+}$ to 0.435 V (DMSO).[4] Spectroelectrochemistry measurements were obtained at ambient temperature with a custom designed three-electrode cell using Hewlett-Packard 8452A diode array spectrophotometer and CHI620E. Elemental analyses were performed by Midwest Microlab, LLC in Indianapolis, IN. Cell culture media consisted of RPMI 1640 with 2 mM glutamine and 25 mM HEPES (Corning 10041CV) with 10% heat-inactivated fetal bovine serum (Sigma f6178) and 1X penicillin-streptomycin (Sigma p4333). Trypsin (Hyclone SH30236.01) and Dulbecco's Phosphate Buffer Saline (Sigma R5886) were used for general cell maintenance and harvesting. Cell lines were obtained from the ATCC (A549 and PC-3) and Prof. Zahid Siddik at MD Anderson (A2780 and A2780CP). Thiazolyl blue tetrazolium bromide (Alfa Aesar L11939) was used for cell proliferation assays. Cell culture plastic ware consisted of generic T-75 flasks, 80.5 mm diameter culture dishes, and treated 96-well plates. Zebrafish experiments were approved (I13009) and performed in compliance with the relevant laws and institutional guidelines set forth by the Animal Care and Use Committee (IACUC).

Synthesis

[Bis(1,3-dimesityl-4,5-naphthoquino-imidazol-2-ylidene)-gold(I)] [Silver(I) dichloride], Compound 1.

(1,3-Dimesitylnaphthoquinimidazol-2-ylidene)-silver chloride (117.4 mg, 0.203 mmol, 1.0 eq) and (C_4H_8S)AuCl (29.3 mg, 0.0914 mmol, 0.45 eq) were combined in a 20 mL scintillation vial with 4 mL THF under an inert atmosphere. After stirring at 60°C for 16 h, the mixture was decanted, and the dark solid was washed with 3 x 5 mL of Et_2O to reveal a yellow product. This was dissolved in minimal CH_2Cl_2 and filtered through a plug of Celite into a pre-weighed 20 mL scintillation vial; the solvent was removed under reduced pressure. Yield: 82.1%. ^1H NMR (δ, CD_2Cl_2, 300 MHz): 1.63 (s, 24H, Mes), 2.43 (s, 12H, Mes), 6.97 (s 8H, Mes), 7.71-7.77 (m, 4H, NQ), 7.95-8.00 (m, 4H, NQ). ^{13}C NMR (δ, CD_2Cl_2, 75 MHz): 17.6, 21.6, 127.7, 129.9, 132.3, 132.5, 132.7, 134.4, 135.6, 141.0, 174.4, 192.6. HRMS (ESI) for [$C_{58}H_{52}N_4O_4Au$]$^+$ [M]$^+$ Calcd. 1065.3654 Found 1065.3656. Anal. Calcd. for: $C_{58}H_{52}N_4O_4AuAgCl_2$: C, 55.96; H, 4.21; N, 4.50; Found: C, 56.48; H, 4.34; N, 4.56. IR (cm^{-1}): 1681 (KBr).

[Bis(1,3-dimesityl-4,5-naphthoquino-imidazol-2-ylidene)-gold(I) Chloride, Compound 2.

1,3-Dimesitylnaphthoquinimidazolium chloride (85.2 mg, 0.1809 mmol, 1.0 eq) was added to NaHMDS (33.2 mg, 0.1809 mmol, 1.0 eq) in a 20 mL scintillation vial and stirred at 25°C for 16 h in 2 mL of toluene. The resulting mixture was filtered through a plug of Celite into a pre-weighed 20 mL scintillation vial containing (C_4H_8S)AuCl (26.1 mg, 0.081 mmol, 0.45 eq) and stirred at 25°C for thirty minutes. The dark precipitate was subjected to a series of washes (2 × 4 mL of toluene and then 3 x 4 mL of Et_2O) to yield a yellow solid (67.0 mg). Yield: 74.7%. ^{1}H NMR (δ, CD_2Cl_2, 300 MHz): 1.74 (s, 24H, Mes), 2.55 (s, 12H, Mes), 7.08 (s 8H, Mes), 7.83-7.89 (m, 4H, NQ), 8.06-8.12 (m, 4H, NQ). ^{13}C NMR (δ, CD_2Cl_2, 75 MHz): 17.6, 21.6, 127.7, 129.9, 132.2, 132.5, 132.7, 134.4, 135.6, 141.0, 174.4, 192.6. HRMS (ESI) for [$C_{58}H_{52}N_4O_4Au$] $[M]^+$ Calcd. 1065.3654 Found 1065.3665. Anal. Calcd. for: $C_{58}H_{52}N_4O_4AuCl$: C, 63.24; H, 4.76; N, 5.09; Found: C, 63.33; H, 4.73; N, 5.05. IR (cm^{-1}): 1681 (KBr).

(1,3-Dimesityl-4,5-naphthoquino-imidazol-2-ylidene)-gold(I) Chloride, Compound 3.

(1,3-Dimesitylnaphthoquinimidazol-2-ylidene)-silver chloride (119.4 mg, 0.206 mmol, 1.0 eq) and (C_4H_8S)AuCl (66.1 mg, 0.206 mmol, 1 eq) were combined in a 20 mL scintillation vial with 4 mL THF under an inert atmosphere. After stirring at 40°C for 5 h, the opaque reaction mixture was stirred for an additional 12 h at 25°C. This was then filtered through a plug of Celite into a pre-weighed 20 mL scintillation vial, and the solvent was removed under reduced pressure. The resulting solid was washed successfully with 3 x 5 mL of Et_2O to reveal a yellow product. Yield: 67.8%. ^{1}H NMR (δ, CD_2Cl_2, 300 MHz): 2.15 (s, 12H, Mes), 2.49 (s, 6H, Mes), 7.20 (s 4H, Mes), 7.83-7.89 (m, 4H, NQ), 8.11-8.16 (m, 4H, NQ). ^{13}C NMR (δ, CD_2Cl_2, 75 MHz): 18.2, 21.5, 127.5, 130.0, 132.3, 132.4, 133.4, 134.6, 135.3, 141.1, 174.6, 183.4. HRMS (ESI) for [$C_{29}H_{26}N_2O_2AuCl$] $[M + Na]^+$ Calcd. 689.1246 Found 689.1230. Anal. Calcd. for: $C_{29}H_{26}N_2O_2AuCl$: C, 52.23; H, 3.93; N, 4.20; Found: C, 51.98; H, 3.85; N, 4.12. IR (cm^{-1}): 1679 (KBr).

Crystal Structure

For single crystal X-ray examination and data collection, suitable crystals were mounted in a loop with Paratone-N oil and transferred to the goniostat bathed in a cold stream. Intensity data were collected at 150K on a standard Bruker DUO APEX-II CCD diffractometer using graphite-monochromated Mo Kα radiation, λ = 0.71073 Å. For compound **1**, the data collection frames were measured for a duration of 5-s at 0.5^o intervals of ω with a maximum θ value of ~28°. The data frames were processed using the program SAINT.[5] The data were corrected for decay, Lorentz and polarization effects as well as absorption and beam corrections based on the multi-scan technique contained in SADABS.[6]

The structures were solved SHELXT[7] and SHELXL[8] based on F^2. Non-hydrogen atoms were refined with anisotropic displacement parameters. H-atoms were calculated and treated with a riding model. The H-atom isotropic displacement parameters were defined as a*U_{eq} of the adjacent atom (a = 1.5 for methyls, 1.2 for all others). For compound **1**, the refinement converged with crystallographic agreement factors of R1 = 2.3%, wR2 = 6.4% for 10778 reflections with I>2σ(I) (R1 = 2.82%, wR2 = 6.65% for all data) and 170 variable parameters.

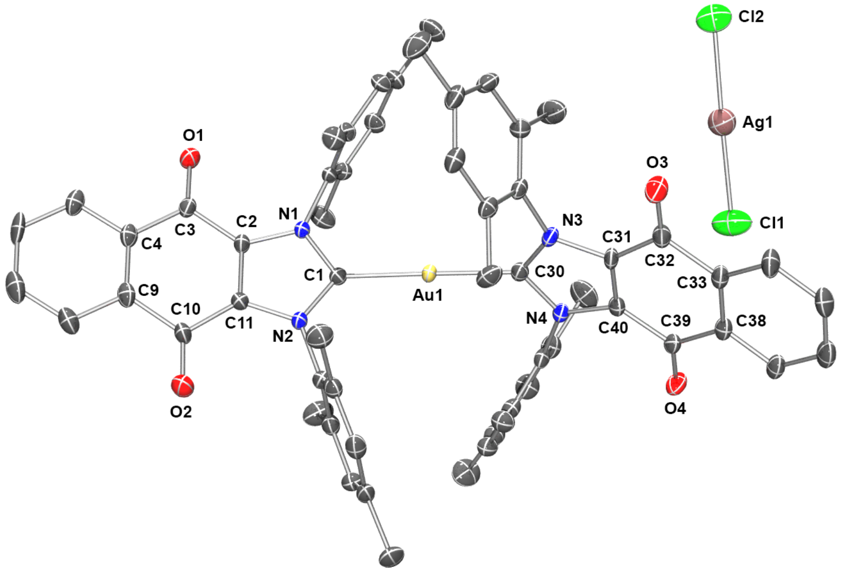

Figure 4 (Taken from article text, but placed in ESI to provide clarity to **Table S1**): Molecular Structure of compound **1** drawn using POV-Ray. Thermal ellipsoid plots are drawn at 50% probability level and hydrogen atoms are omitted for clarity. Selected bond lengths (Å) and angles (deg): C1–N1, 1.347(6); C1–N2, 1.359(6); C30–N3, 1.356(6); C30–N4, 1.360(6); C1–Au1, 2.012(5); C30–Au1, 2.009(5); C3–O1, 1.217(6); C10–O2, 1.219(6); C39–O4, 1.207(6); C32–O3, 1.221(6); C11–C2, 1.350(7); C40–C31, 1.365(7); Cl1–Ag1, 2.3210(19); Cl2–Ag1, 2.3325(17); N1– C1–N2, 106.4(4); N3–C30–N4, 105.9(4); C1–Au1–C30, 172.87(19); Cl1–Ag1–Cl2, 178.46(8).

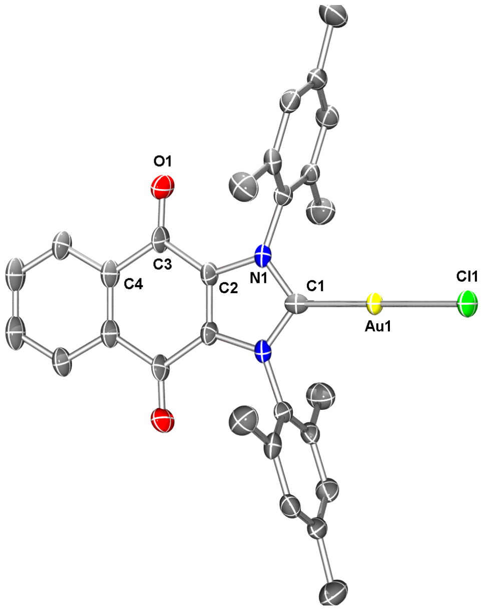

Figure S1: Molecular Structure of compound **3** drawn using POV-Ray. Thermal ellipsoid plots are drawn at 50% probability level and hydrogen atoms are omitted for clarity. Selected bond lengths (Å) and angles (deg): C1–N1, 1.375(10); C2–N1, 1.390(10); C1–Au1, 1.944(12); C3–O1, 1.217(6); C2–C2', 1.365(17); Au1–Cl1, 2.272(3); N1–C1–N1', 104.9(10); C1–Au1–Cl1, 177.3(4).

Table S1. Crystallographic and refinement data.

	Compound **1**	Compound **3**
CCDC	1520307	1537344
solvent	none	used squeeze
formula	$C_{58}H_{52}N_4O_4AuAgCl2$	$C_{29}H_{26}AuClN_2O_2$
fw	1244.7	666.95
xtl system	orthorhombic	tetragonal
space grp	*Pna*2(1)	*I-42m*
color, habit	yellow, rod	Yellow, block
a, Å	22.040(3)	19.1131(19)
b, Å	16.674(2)	19.1131(19)
c, Å	14.244(2)	14.8158(16)
α, deg.	90.00	90.00
β, deg.	90.00	90.00
γ, deg.	90.00	90.00
V, Å^3	5234.7(11)	5412.37
T, K	150(2)	171K
Z	4	8
R1, wR2[a]	0.023, 0.064	0.0292, 0.0618
GoF on F^2	1.049	1.072

[a] $R1 = \Sigma||F_o| - |F_c||/\Sigma|F_o|$. [b] $R_w = \{[\Sigma w(F_o^2 - F_c^2)/\Sigma w(F_o^2)^2\}^{1/2}$; $w = 1/[\sigma^2(F_o^2) + (xP)^2]$, where $P = (F_o^2 + 2F_c^2)/3$.

Electrochemical Studies

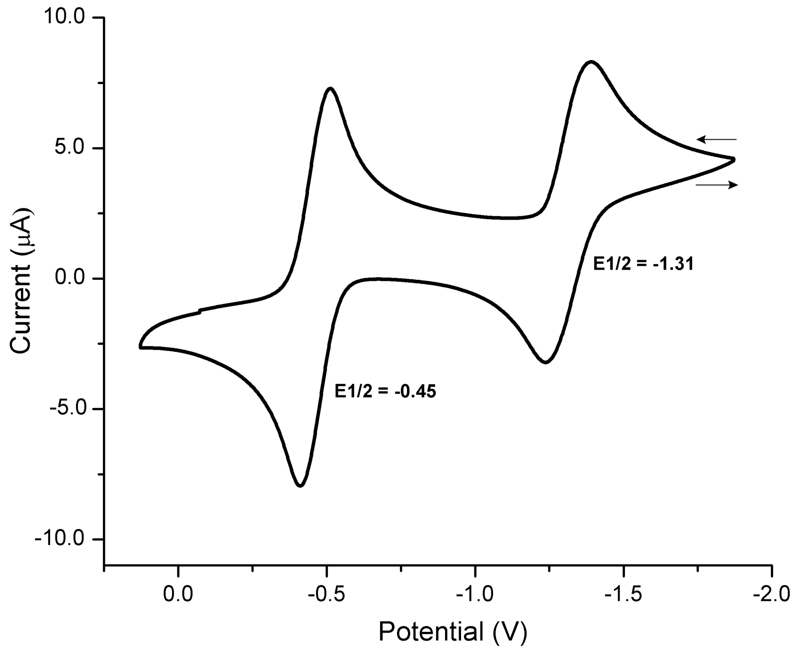

Figure S2: Cyclic voltammogram of compound **1** with 0.1 M [N(*n*Bu)][PF_6] in DMSO (1 mM) as referenced to SCE (internal standard $Fc^{0/+}$, adjusted to 0.435 V vs. SCE).

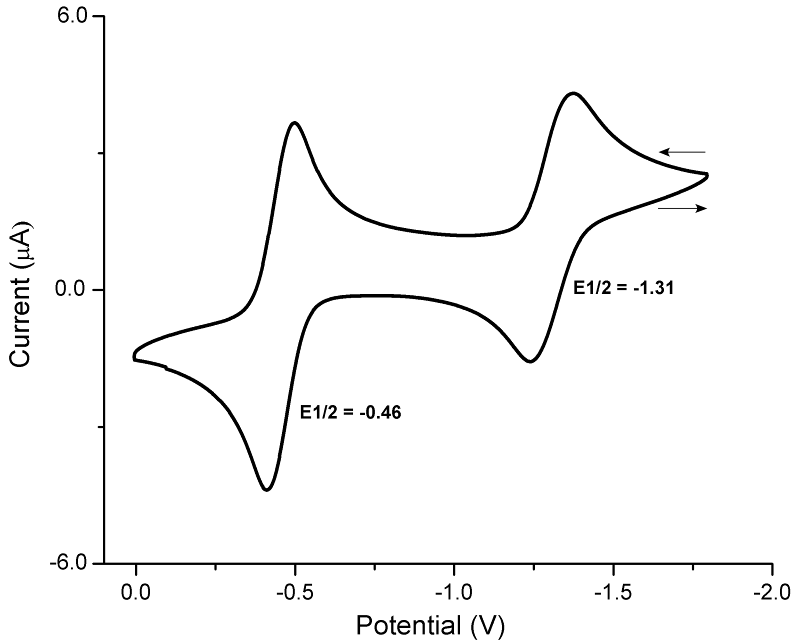

Figure S3: Cyclic voltammogram of compound **2** with 0.1 M [N(*n*Bu)][PF_6] in DMSO (1 mM) as referenced to SCE (internal standard $Fc^{0/+}$, adjusted to 0.435 V vs. SCE).

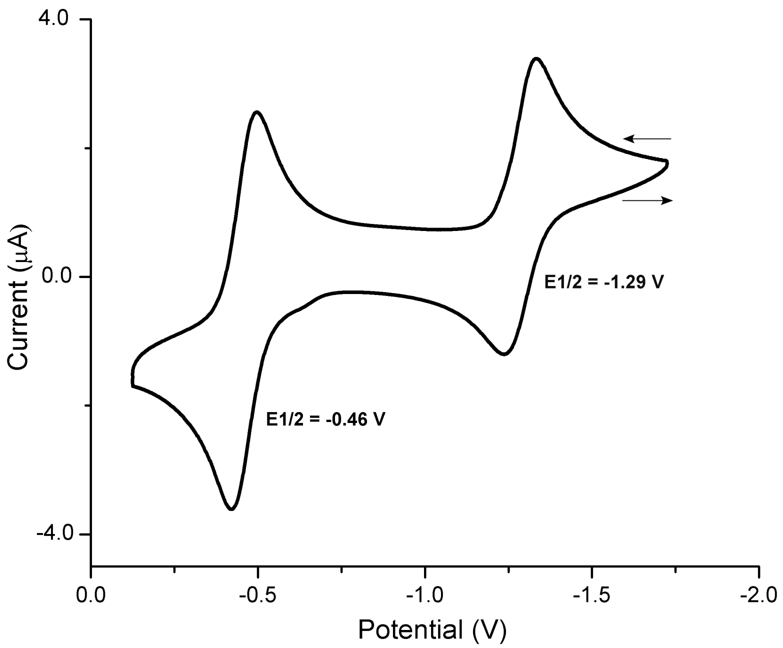

Figure S4: Cyclic voltammogram of compound **3** with 0.1 M [N(*n*Bu)][PF_6] in DMSO (1 mM) as referenced to SCE (internal standard $Fc^{0/+}$, adjusted to 0.435 V vs. SCE).

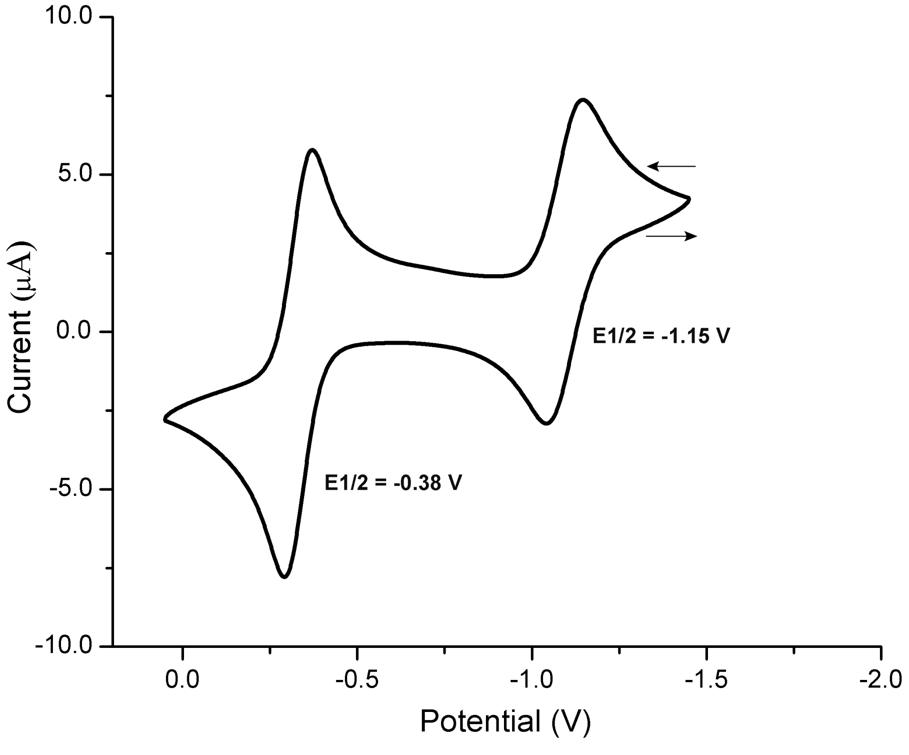

Figure S5: Cyclic voltammogram of compound **4**[**H**][**Cl**] with 0.1 M [N(^{n}Bu)][PF_6] in DMSO (1 mM) as referenced to SCE (internal standard $Fc^{0/+}$, adjusted to 0.435 V vs. SCE).

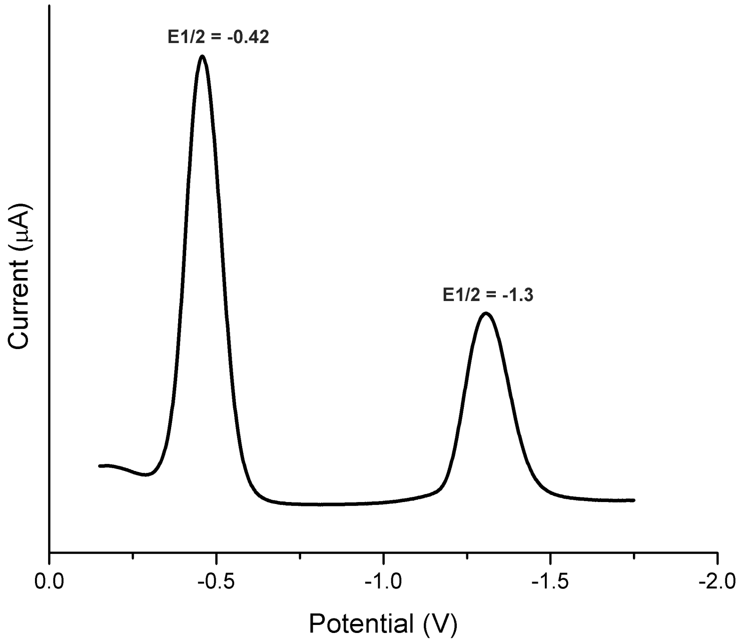

Figure S6: Differential pulse voltammogram of compound **1** with 0.1 M [N(^{n}Bu)][PF_6] in DMSO (1 mM) as referenced to SCE (internal standard $Fc^{0/+}$, adjusted to 0.435 V vs. SCE).

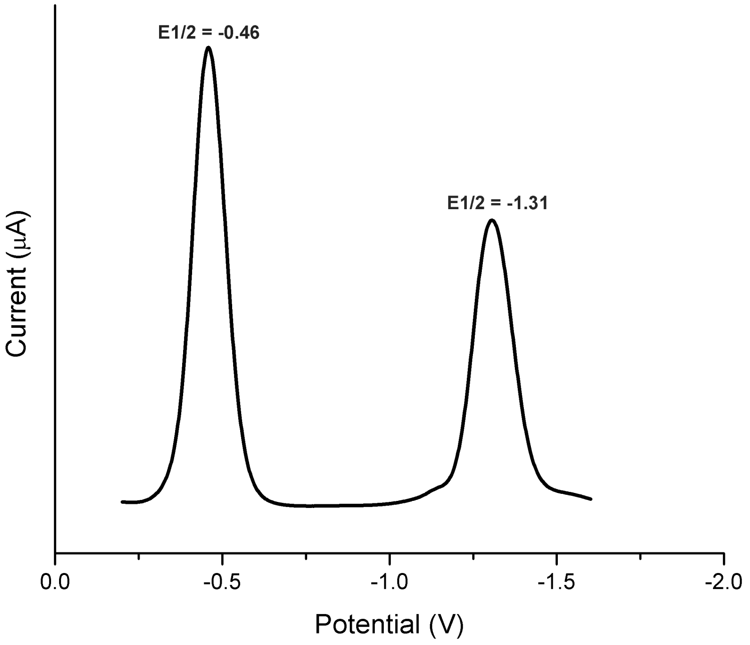

Figure S7: Differential pulse voltammogram of compound **2** with 0.1 M [N(^{n}Bu)][PF_6] in DMSO (1 mM) as referenced to SCE (internal standard $Fc^{0/+}$, adjusted to 0.435 V vs. SCE).

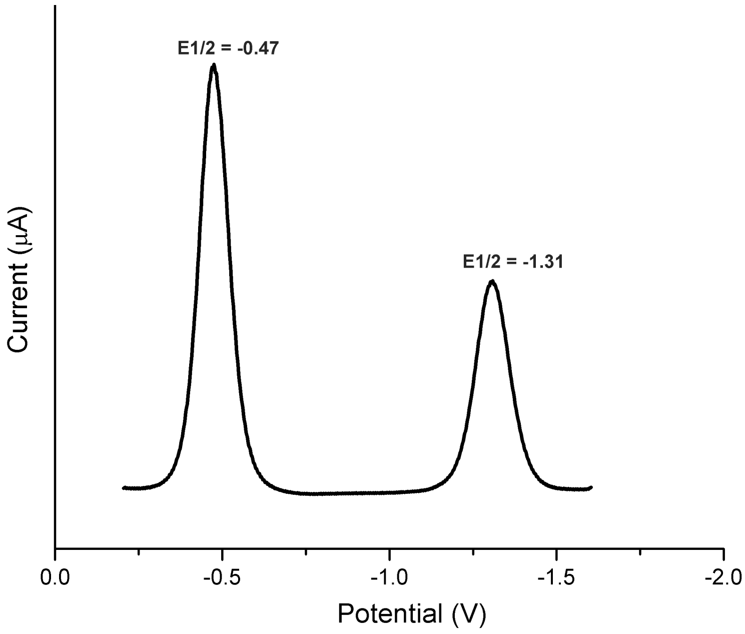

Figure S8: Differential pulse voltammogram of compound **3** with 0.1 M [N(^{n}Bu)][PF_6] in DMSO (1 mM) as referenced to SCE (internal standard $Fc^{0/+}$, adjusted to 0.435 V vs. SCE).

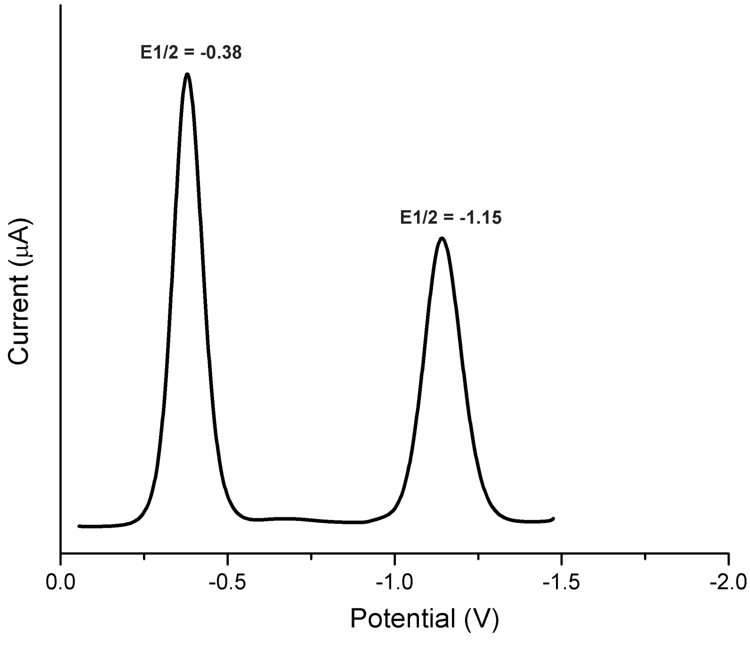

Figure S9: Differential pulse voltammogram of compound **4**[**H**][**Cl**] with 0.1 M [N(^{n}Bu)][PF_6] in DMSO (1 mM) as referenced to SCE (internal standard $Fc^{0/+}$, adjusted to 0.435 V vs. SCE).

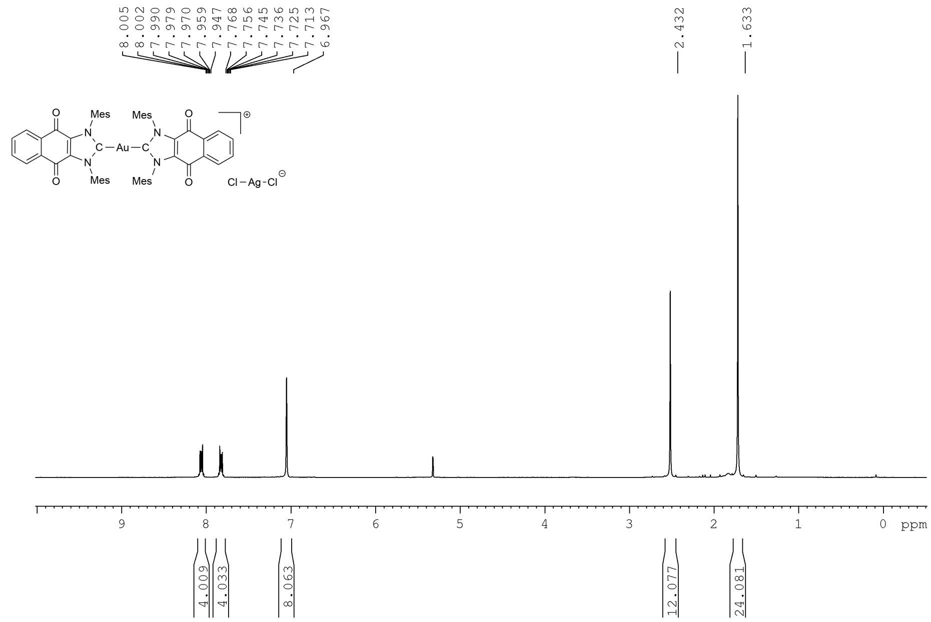

Figure S10: ^{1}H NMR spectrum of compound **1.**

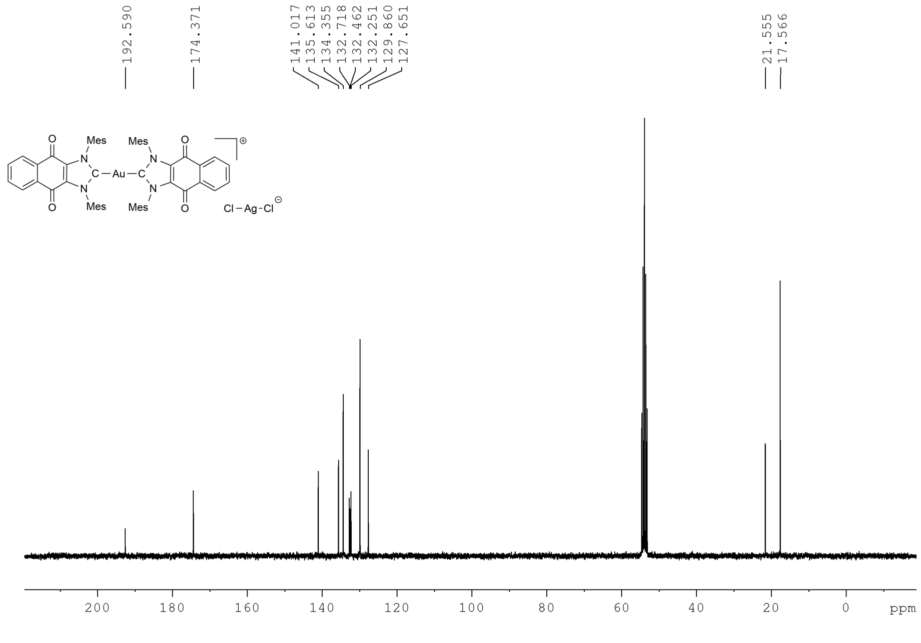

Figure S11: ^{13}C NMR spectrum of compound **1.**

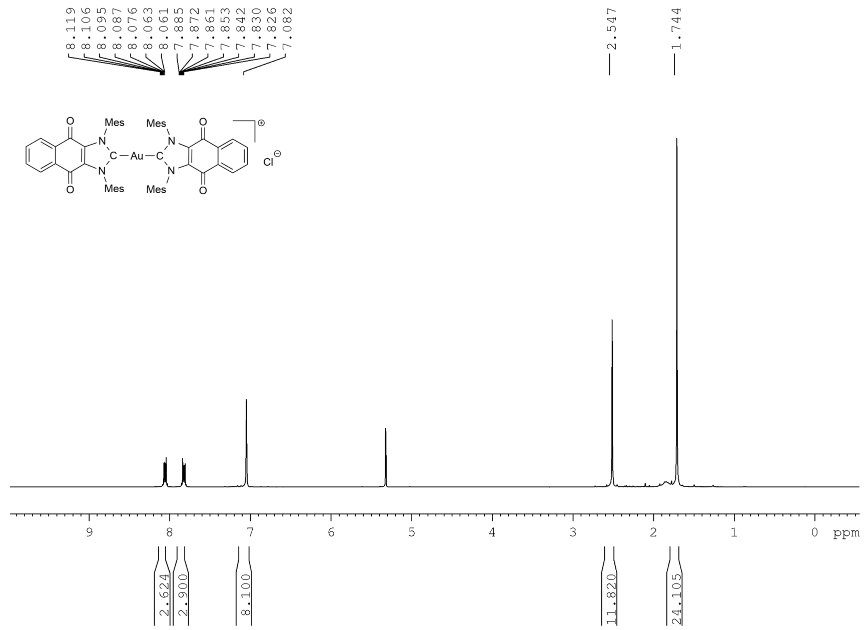

Figure S12: ^{1}H NMR spectrum of compound **2.**

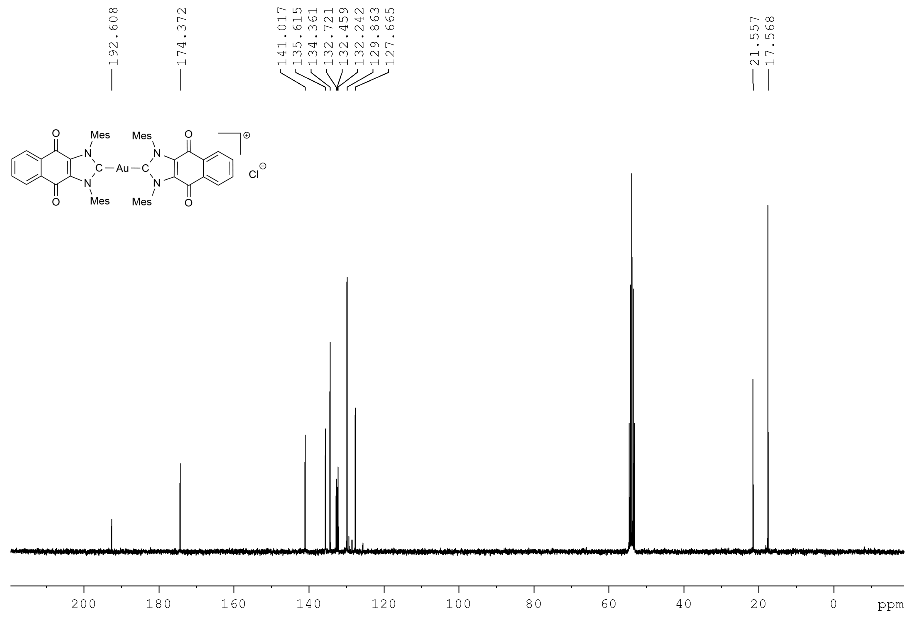

Figure S13: ^{13}C NMR spectrum of compound **2.**

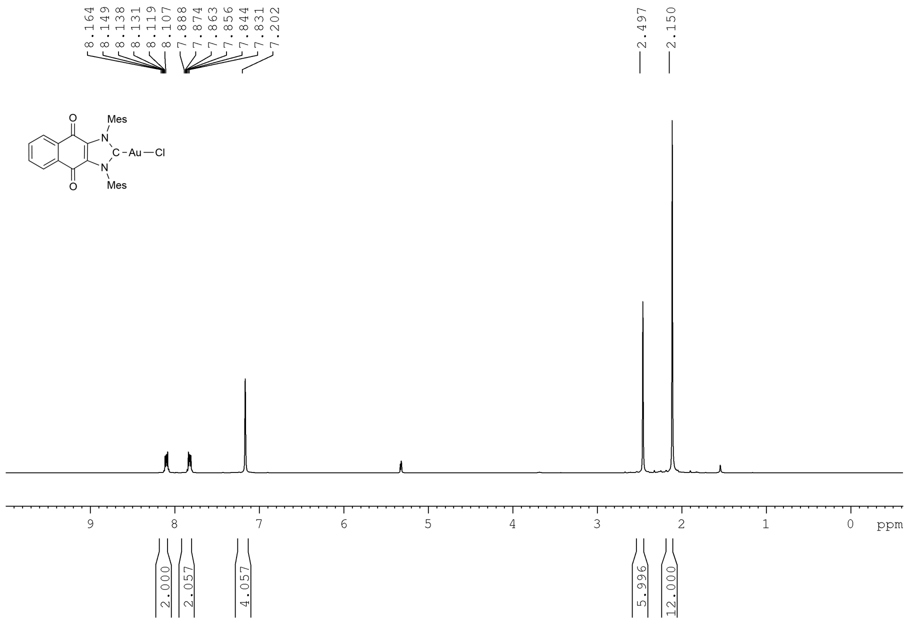

Figure S14: ^{1}H NMR spectrum of compound **3**.

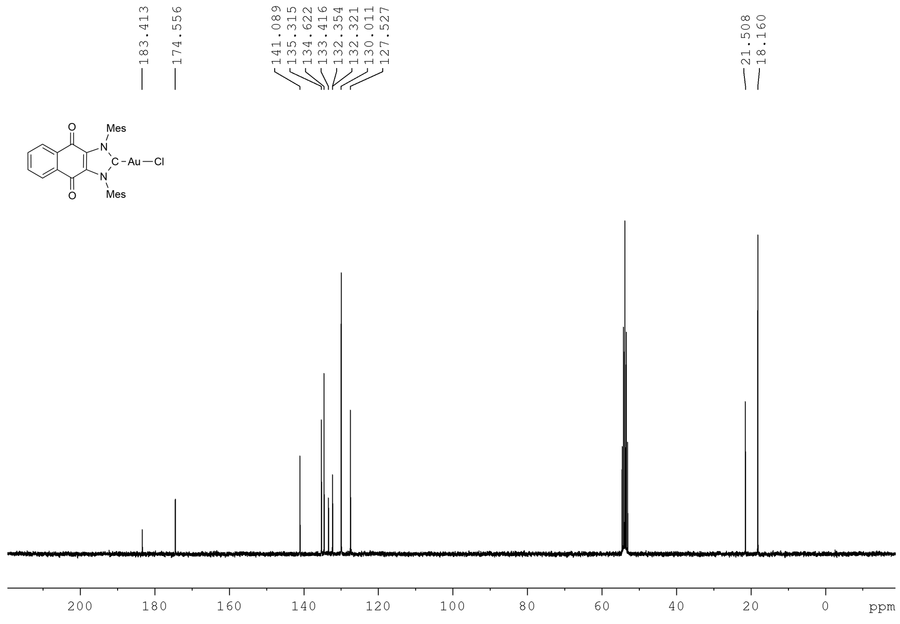

Figure S15: ^{13}C NMR spectrum of compound **3**.

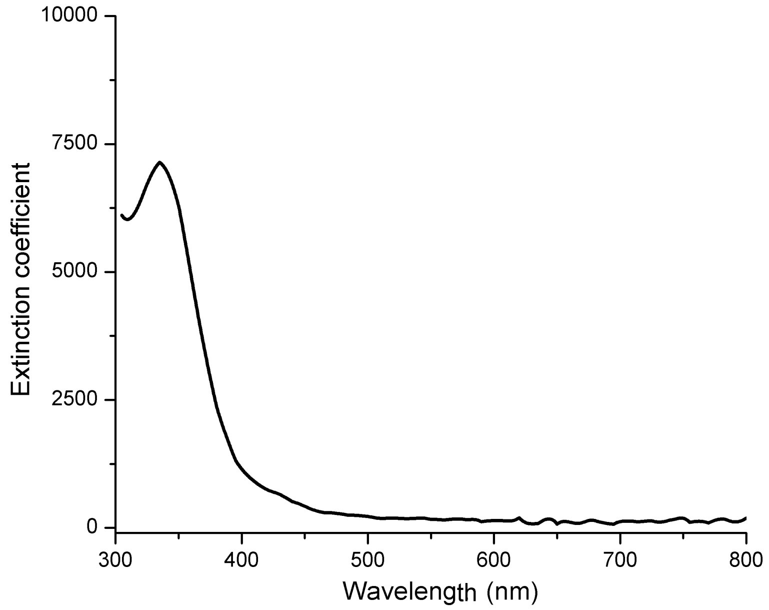

Figure S16: Electronic absorption spectra of compound **2** recorded in DMSO.

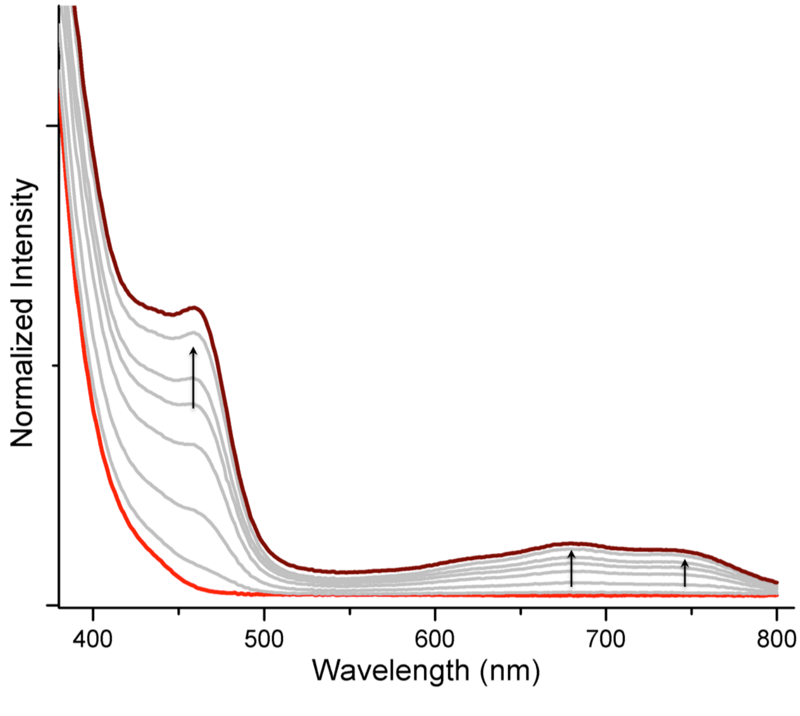

Figure S17: Electronic absorption spectra recorded during bulk reduction of compound **2** ([bis(1,3-dimesityl-4,5-naphthoquino-imidazol-2-ylidene)-gold(I) chloride) (NQ $\rightarrow$ NQ^{2-}) holding at -1.5 V (vs. AgCl) in DMSO with 0.1 M $[N(nBu)_4][PF_6]$ as the supporting electrolyte at 25 °C. The arrows indicate the direction of the spectral change over time.

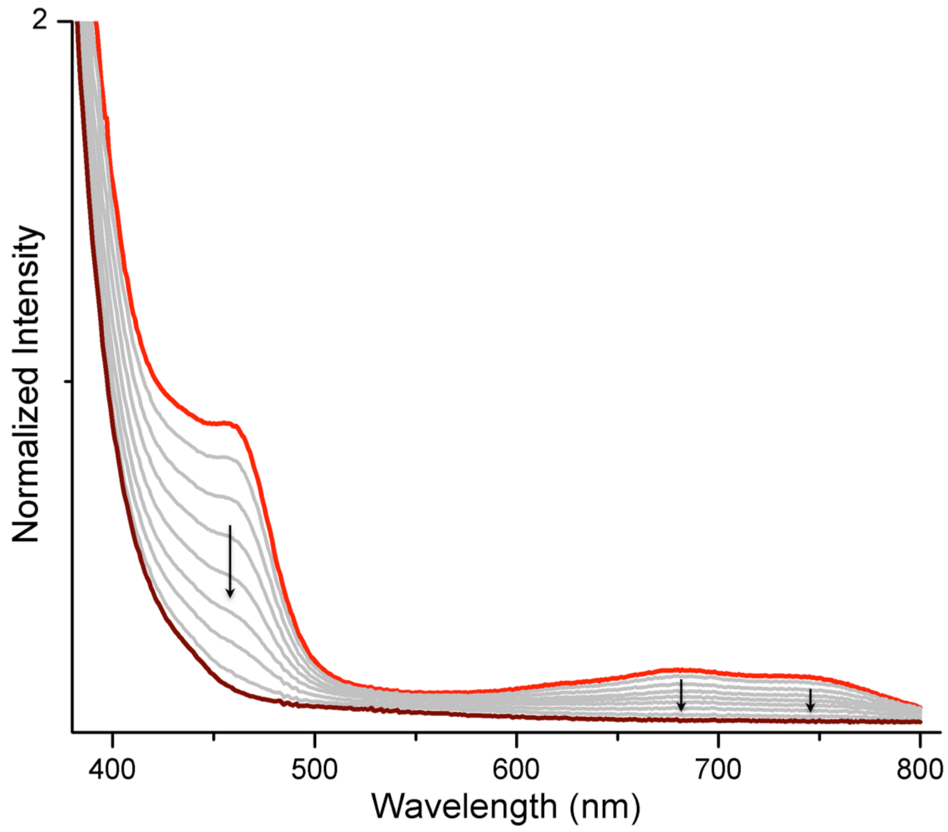

Figure S18: Electronic absorption spectra recorded during bulk oxidation of reduced compound **2** ($NQ^{2-} \rightarrow NQ$) holding at -0.1 V (vs. AgCl) in DMSO with 0.1 M $[N(nBu)_4][PF_6]$ as the supporting electrolyte at 25 °C. The arrows indicate the direction of the spectral change over time.

Biological preparations and assays

Experimental compound preparation. Stock solutions of complexes **1**–**3**, **4**[H][Cl], auranofin (Tocris Bioscience 4600), and doxorubicin (Tocris Bioscience 2252) were dissolved in DMSO (2.5–10 mM, depending on the compound and its solubility), aliquoted, and stored at -80 °C prior to use.

Biological Materials and Cell Lines. Cell lines were obtained from the ATCC (A549 and PC-3) and Prof. Zahid Siddik at M.D. Anderson Cancer Center (A2780 and A2780CP). Cell culture plastic ware (i.e. T-75 flasks, treated 6-well plates, treated 96-well plates, and cell scrapers) were obtained from Fisher Scientific. Cell were cultured in RPMI 1640 culture medium (+2mM glutamine, +25mM HEPES, Corning 10041cv) supplemented with 10% heat-inactivated fetal bovine serum (RMBIO FBS-BBT) and 1X penicillin-streptomycin (Sigma p4333). Unless otherwise noted, incubators were set to 37°C and 5% CO_2. Trypsin (HyClone SH30236.01) and 1X Phosphate Buffered Saline (HyClone SH30258.02, diluted in autoclaved deionized water) were utilized for general cell line maintenance and harvesting. Trace Metal Grade HCl (Fisher A508-4) and HNO_3 (Sigma 225711-475mL) and autoclaved deionized water were used for studies involving ICP-MS. Diluent for samples and standards run on the ICP-MS was a 3% HCl solution made with the Trace Metal Grade HCl and autoclaved deionized water. The activity of thioredoxin reductase was monitored in live cells using a solution of lipoate (Tokyo Chemical Industry Co., Ltd. L0207) and DTNB (Acros Organics 117540050) in 1X HBSS (Life tech 14025-092).

Cell Proliferation Studies. Cells were harvested and seeded into 96-well culture plates (Costar 07-200-90) in 100μL of culture media. They were allowed to incubate overnight at 37 °C in the presence of 5% CO_2. A549 was seeded at a density of 1000 cells/well, A2780 at 2500 cells/well, A2780CP at 3000 cells/well, and PC-3 at 2000 cells/well. The next day, appropriate serial dilutions of drug stocks in culture media were made. To each well of a 96 well plate was added 100 μL of the appropriate solution. After a total of three days, a 50 mL aliquot of 3 mg/mL tetrazolium dye, 3-(4,5-dimethylthiazol-2-yl)-2,5-diphenyltetrazolium bromide (Alfa Aesar L11939) was added to each well, followed by a 4 hr incubation period at 37 °C. The medium was then removed, the resulting formazan was dissolved in 50 mL DMSO and the respective absorbances were measured at 560–650 nm using a microplate reader (Molecular Devices, Sunnyvale, CA). Absorbance values were corrected for background and then normalized to wells containing untreated cells to allow for plate-to-plate comparisons. The data are shown as mean inhibition of proliferation or growth as a percentage of control cells and are from 2–3 replicate experiments. Resulting dose response curves were subjected to linear regression analysis (Origin by OriginLab, Inc.) for determination of IC_{50} values.

ICP-MS Analysis and Preparations. Roughly 7–8 x10^6 cells were grown to 70-80% confluence in T-75 cell culture flasks (Thermo 130190) and treated with respective compound in RPMI medium supplemented with 10% FBS and antibiotics for the desired incubation time. The medium was then removed and cells were washed with 8 mL 1X warm PBS. 2 mL 1X PBS was then added to each flask, and the cells were removed by scraping. Each cell suspension was then pipetted into a clean, labeled 15 mL centrifuge tube (Thermo 339650). Cells were pelleted by centrifuging at 1,000 rpm for 5 min in an Eppendorf 5804 with a swing-bucket rotor. The supernatant was removed by vacuum aspiration, and the pellet was resuspended in 50 μL autoclaved deionized water. Suspension/lysates were then kept at -80 °C until further use. After thawing, the lysates were then sonicated using a Branson Digital Sonifier, and 5 μL aliquots were taken for determination of protein content of the sample. Lysates were digested with 80 μL aqua regia and heated to 65 °C for 1 h. Digested lysates were diluted with 4 mL 3% Trace Metal Grade HCl and gold content was measured on a Perkin Elmer NexION 300X set to detect gold at 197 m/z. Gold concentrations were quantified using the linear range of a standard curve generated by serial dilution of an ICP-MS gold standard (Ricca MSAU1KH-100) in 3% HCl. Values were then normalized to protein

content as determined using a Modified Lowry Protein Assay Kit (Thermo 23240) and known concentrations of bovine serum albumin (Fisher 9048-46-8).

The Modified Lowry Assay was used to determine the protein content of each sample for normalization purposes. The standard curve for each run was made from a 2-fold serial dilution of fresh 5 mg/mL BSA solution. Aliquots were diluted to within the linear range of the standard curve using autoclaved DI water, and subjected to the Modified Lowry Assay in round-bottomed 96-well clear plates (Fisher 12565500). Each well contained 10 µL of sample, 50 µL of Modified Lowry Reagent, and 5 µL of 1 N Folin-Ciocalteau Reagent. All other aspects of the procedure were as detailed in the microplate procedure instructions that come with the kit.

Serum Protein Binding Studies. The binding of gold from each compound to proteins in non-heat inactivated fetal bovine serum (HyClone SH30910.02) was observed during a time-dependent study. A 25 µM solution of each compound in 3mL warm FBS was made. The solutions were kept at 37°C. Over time, 100 µL aliquots of each solution were taken and precipitated into 400 µL cold methanol. Precipitated aliquots were stored at -80°C. The methanolic suspensions were then centrifuged at 11,000rcf for 5 min in an Edvotek microcentrifuge. The supernatants were pipetted into new microcentrifuge tubes. Samples were prepared for ICP-MS by adding 200 µL supernatant from each sample to 2 mL 3% Trace Metal Grade HCl. Time points were at addition of compound (T0), 4, 6.25, 9.75, 12, 24, 48, 72, and 100 h.

Detection of ROS by Flow Cytometry. Tumor cells ($1x10^6$) were plated in a 6-well plate overnight and then incubated with media containing various complexes described at concentrations of 0.625–2.5 µM. Control cells were treated with vehicle only. After a 6 h. incubation, the cells were washed with PBS, detached with 0.250 mL 1X trypsin, collected in a 15 mL conical tube, and pelleted by centrifugation (3 min @ 300 g) at 5 °C. The resulting pellet was again washed 2x with cold PBS and pelleted at 5 °C. The pellet was then suspended in freshly prepared 2 µM CM-H_2DCFDA (Lifetech C6827) in PBS for a final cell concentration of $1x10^6$ cells/mL and incubated in the dark at 37 °C for 15 min. The cells were then pelleted, washed with 2 mL PBS, and re-suspended in PBS containing 2 µg/mL propidium iodide (PI, Chemodex P0023) for a final cell concentration of $0.5x10^6$ cells/mL. Control samples of unstained cells, cells stained with only CM-H_2DCFDA, and cells stained with only PI were also prepared. Each sample was added to one well of a 96-well plate. Samples were then subjected to FACS analysis using a Millipore Guava easyCyte 8 and analyzed using the Guava inCyte software.

Fluorescence Microscopy. Tumor cells were harvested and seeded at a density of $2x10^5$ cells/dish in 35 mm dishes containing a poly-D lysine coated 10 mm glass diameter (Mat Tek P35GC-1.5-10-C) overnight. Cells were then incubated with 1.25 µM complex at 37 °C 4 h. Post incubation, the media was removed and cells were washed (2x) with PBS. Cells were then incubated with 1 µM CM-H_2DCFDA (Lifetech C6827) in PBS at 37 °C for 15 min. The dye-PBS solution was removed and cells were washed with PBS (2x). To the cells was added a PBS solution containing 1ug/mL Hoechst 33342 (Lifetech H1399) and 50 nM Mitotracker Red FM (Lifetech M22425) for 30 min at 37 °C. After incubation, the dye-PBS solution was removed and cells were washed with PBS (2x). Cells were then imaged fluorescently on a Leica SP5 X White light laser confocal microscope. Images were taken with a 63X, NA 1.4 objective.

Lipoate Inhibition Assays. Cells were harvested and seeded at a density of 10,000 cells/well A549 in 96-well culture plates. They were then incubated overnight at 37°C and 5% CO_2. The next day, appropriate serial dilutions of drug stock in culture media were performed, and 100 µL of the appropriate solution were added to each well. Cells were returned to the incubator for 6 h. Afterwards, the liquid was then removed from each well by vacuum aspiration and replaced by 100 µL of 5 mM lipoate (Tokyo Chemical Industry Co., Ltd. L0207) and 1 mM DTNB (Acros Organics 117540050) in HBSS (Life tech 14025-092). The absorbance of each well at 605 nm was recorded immediately and once every 20 min for three h on

a microplate reader (Molecular Devices, Sunnyvale, CA). Plates were covered with aluminum foil between readings.

Detection of Apoptosis. Tumor cells (2-3 x 10^5) were plated overnight and then incubated with media containing doxorubicin and **1** drug concentrations at 1.25–20 μM. Control cells were treated with vehicle only. At 24 h, the media was collected and the cells were washed with PBS. The PBS washing was collected and the attached cells were then subjected to 0.5X trypsin (diluted with PBS) for 2 min. All media and washings were collected, pelleted by centrifugation (3 min @ 300 g) and washed twice with cold PBS. The cells were once again pelleted and suspended in Annexin-V binding buffer (BD Biosciences 556547) at a final concentration of 1x10^6 cells/mL. After this, 100 μL of cell suspension was added to 1.5 mL centrifuge tubes, followed by 5 μL each of Annexin V-FITC and propidium iodide (PI, 50 μg/mL) solution. Control samples of unstained cells, cells stained with only Annexin V-FITC, and cells stained with only PI were also prepared. Samples were allowed to incubate in the dark for 15 min at ambient temperature. After incubation, each sample was diluted with 200 μL of Annexin V binding buffer, and the resulting 300 μL sample was added to one well of a 96-well plate. Samples were then subjected to FACS analysis using a Millipore Guava easyCyte 8 and analyzed using Guava inCyte software.

Thermal Denaturation Studies. Thermal denaturation studies were performed to determine the ability of **1**, **4**[H][Cl], and doxorubicin to bind a DNA duplex (5'CCGCAGCCA3'/5'TGGCTGCGG3', Integrated DNA Technologies). Stock solutions of 1.6 mM DNA oligomer were prepared in TE buffer and stored at 5 °C. To form the DNA duplex, equimolar aliquots of each DNA oligomer were combined and annealed at 95 °C for 5 min. The DNA sample was then placed on ice and briefly centrifuged to collect condensation. The DNA was aliquoted into separate 0.6 mL micro-centrifuge tubes and 1 molar equivalent of each **1**, **4**[H][Cl], and doxorubicin was added. To each sample, 12 μL of 1 mM EDTA, 12 μL of 100 mM NaCl, and 12 μL of 20 mM MOPS buffer (pH 7.4) were added and each sample was diluted to a total volume of 120 μL for a final complex and DNA duplex concentration of 5 μM. Samples were transferred to an 8 series microcell (1.0 cm path, 130 μL total volume). The absorbance of each sample was measured using a Shimadzu 2600 UV-vis spectrophotometer enabled with a thermal melt apparatus at a ramp rate of 1 °C/min from 20 °C to 95 °C. The melting temperature was determined mathematically (Origin by OriginLab, Inc.) by calculating the maximum of the first derivative of the sigmoidal melt curve.

Zebrafish Toxicity Analysis. To determine the maximum tolerable dose (MTD) for Complex **1,** we performed zebrafish toxicity analysis. [9-14] Briefly, wild-type zebrafish embryos were exposed to Complex **1** from 24 hpf (hours post fertilization) under standard conditions.[15] Embryos were allowed to grow till 3 dpf (days post fertilization) in 1 ml of solutions containing various concentrations complex **1**. The embryonic development of zebrafish embryos was monitored at 48 hpf and 72 hpf to record the number of dead embryos. Complex **1** was dissolved in DMSO after purification and subsequently resuspended in E3 (embryonic medium) at various concentrations (Figure S29) to assess toxicity in zebrafish embryos. Therefore, our controls were WT (wild-type) embryos in E3 with the same concentrations of DMSO as in complex **1** solutions. Lethality assay was performed at concentrations from 0.5 μM to 7 μM and these studies revealed that complex **1** has a LD_{50} (50% lethal dose) of 0.75 μM. Our study identifies the Maximum Tolerable Dose (MTD) of complex **1** for zebrafish embryos is about 0.5 μM. Complex **1** displayed 100% lethality at concentrations above 1 μM (Figure S29). Therefore, we performed all our tumor xenograft exposure assays at 0.5 μM concentrations.

Biological Data

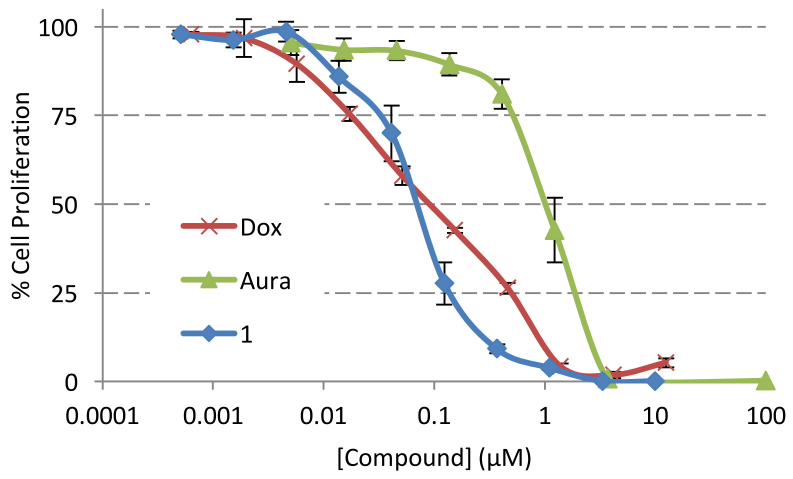

Figure S19: Cell proliferation profile of A549 lung cancer cells treated with **1**, doxorubicin, and auranofin for 72 h. Error bars represent SEM.

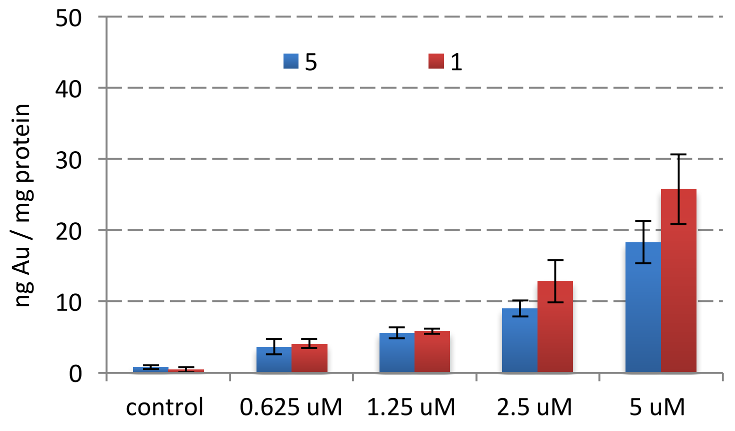

Figure S20: ICP-MS detection of intracellular Au uptake in A549 cells treated in **1** and **5**. Students t-test (unpaired) of **1** (2.5 µM) compared to **5** (2.5 µM) provided p-value >0.2 suggesting no statistical significance. Error bars represent SEM.

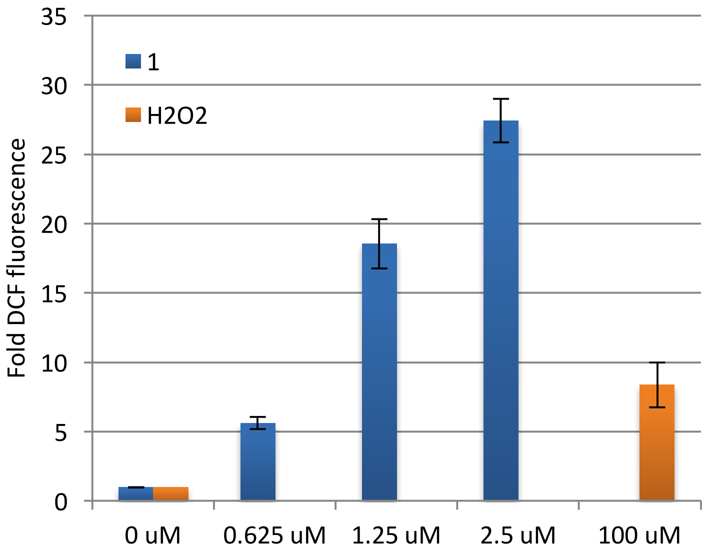

Figure S21: Dose responsive accentuation of intracellular ROS with **1** post 6 h incubation. Error bars represent SEM. A549 treatment with 100 μM H_2O_2 was used as a positive control.

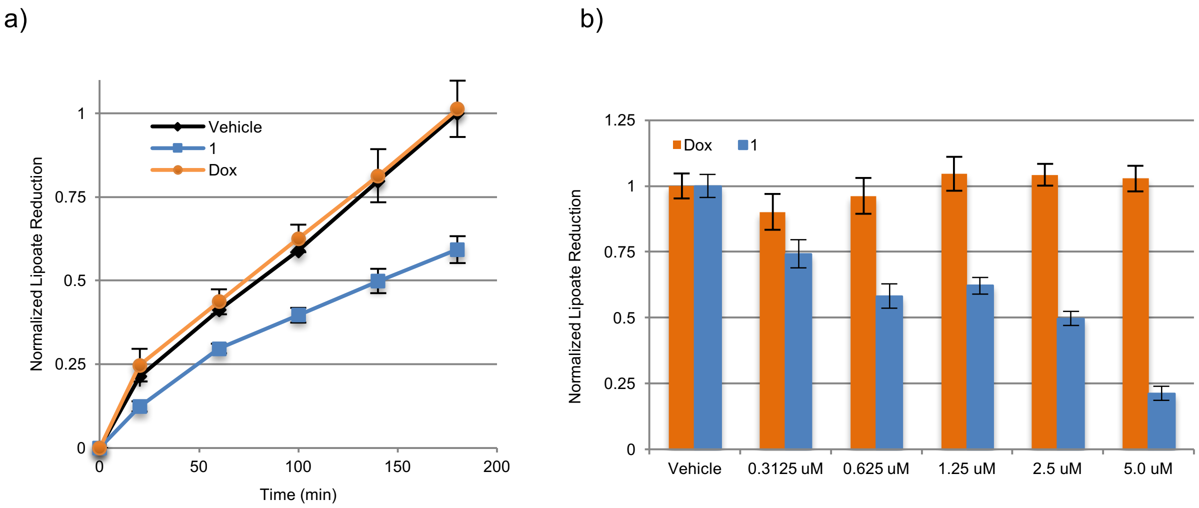

Figure S22: Activity of TrxR as assessed by the detection of lipoate reduction. Comparison in TrxR activity in A549 cells treated with **1** and doxorubicin indicates a time (a) and dose (b) dependent inhibition of enzyme activity by **1** whereas no inhibition of activity in the presence of doxorubicin was detected. Error bars represent SEM.

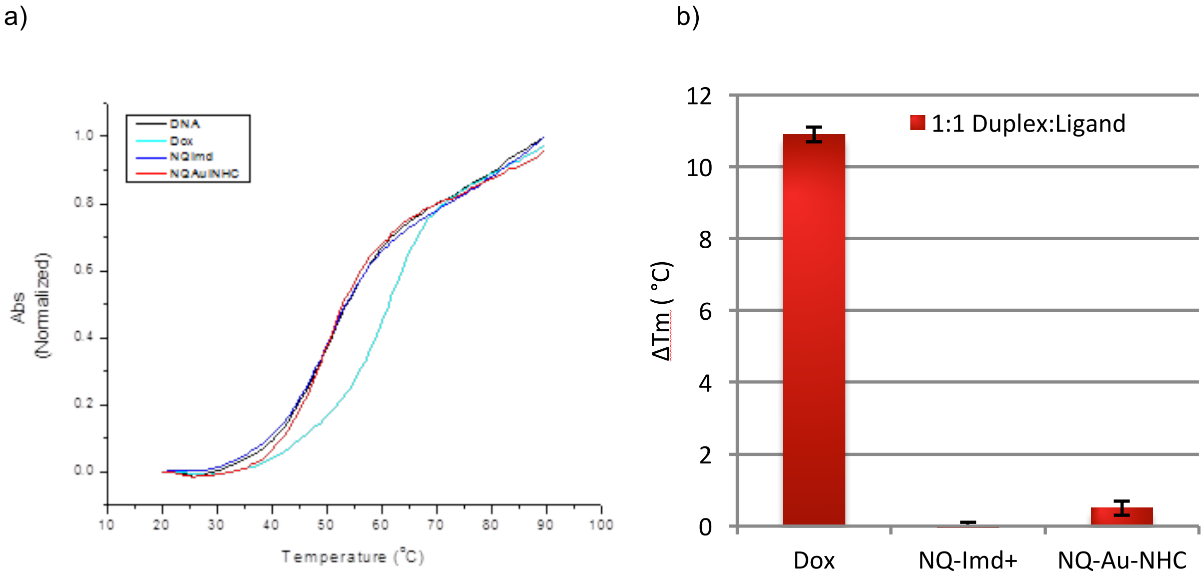

Figure S23: a) Thermal denaturation profiles of a DNA duplex treated with vehicle, doxorubicin, **1** (NQAuNHC), and **4**[H][Cl] (NQimd$^+$). b) Graphical summary of stabilization increase of DNA in the presence of each ligand/complex. Error bars represent SEM.

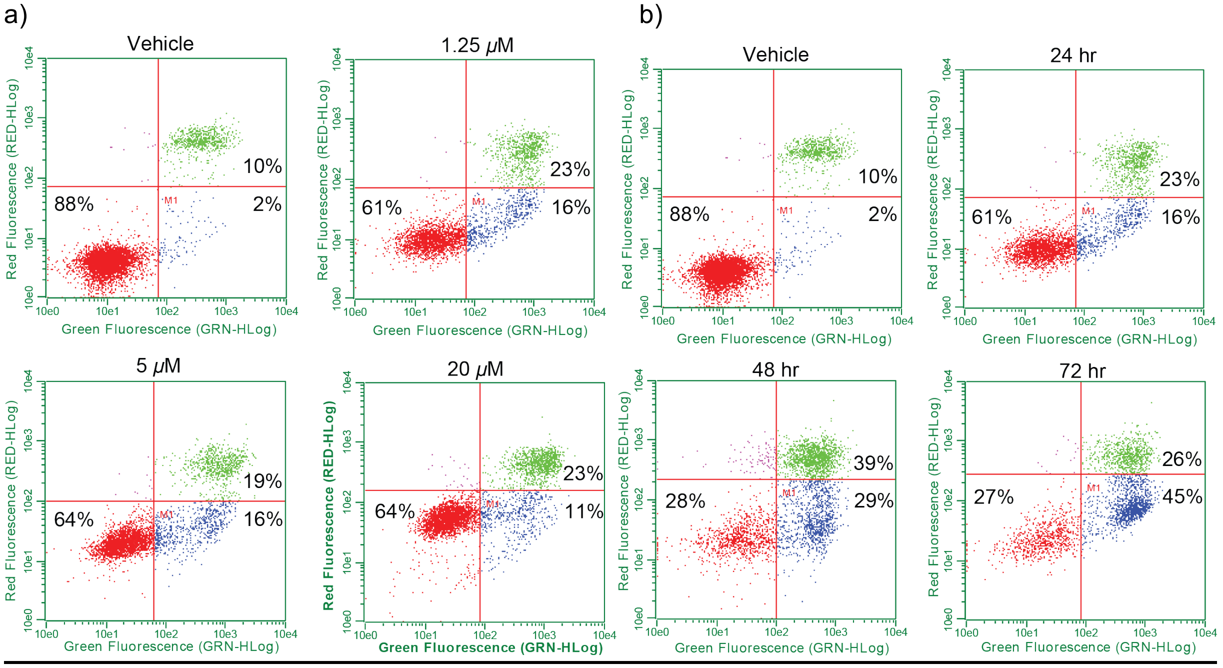

Figure S24: a) Dose dependent induction of apoptosis in A549 cells treated with doxorubicin for 24 h. b). Time dependent induction of apoptosis in A549 cells treated with 1.25 μM doxorubicin.

Comparison of 1 and 2

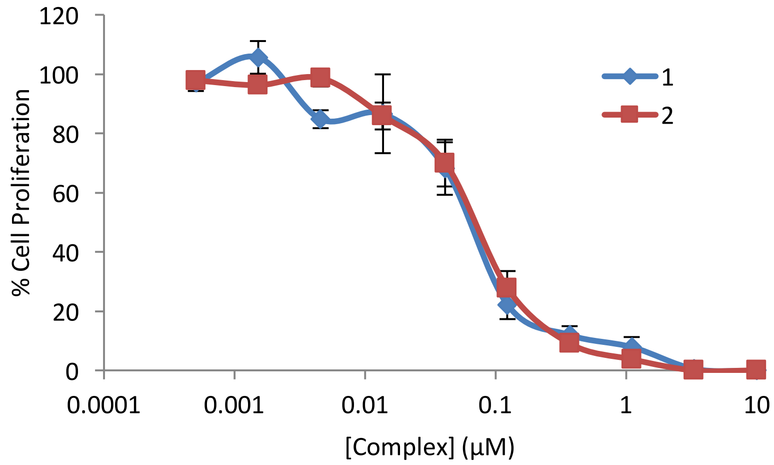

Figure S25: Average percent proliferation of A549 cells exposed to either **1** or **2** for 72 h at varying concentrations. Error bars represent SEM.

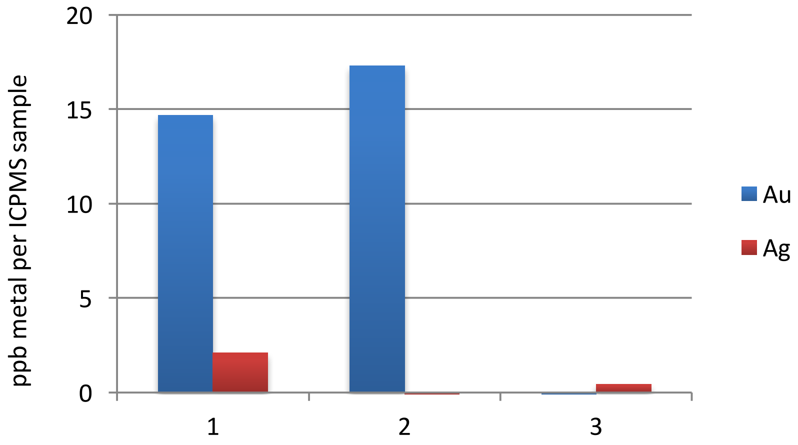

Figure S26: Side by side ICP-MS detection of intracellular Au and Ag concentrations in A549 human lung cancer cells treated with 2.5 μM of complexes **1**–**3** for 6 h. Data illustrates similar Au uptake between **1** and **2**. In addition, the Au:Ag ratio of **1** was found to be 7:1 suggesting that the $AgCl_2$ counterion does not enter the cell and possibly exchanges with other salts in cell culture medium. An Au:Ag ratio of 1:1 would suggest uptake as a complete **1***$AgCl_2$ complex.

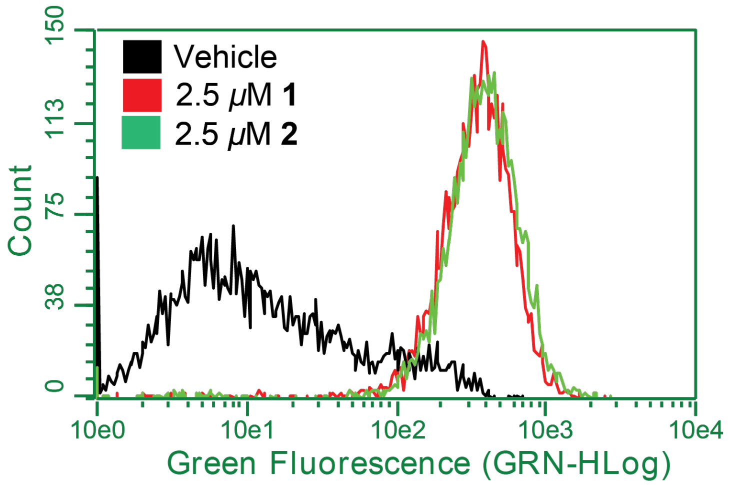

Figure S27: Comparison of intracellular ROS in cells exposed to 2.5 μM either **1** or **2**.

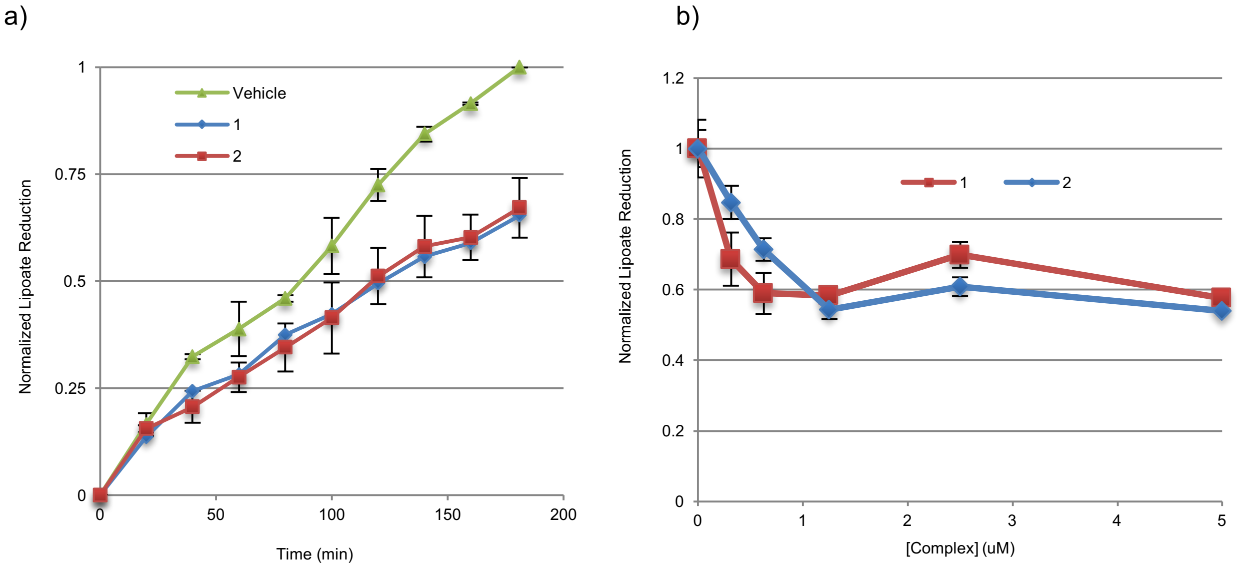

Figure S28: a) Time dependent lipoate reduction in A549 cells exposed to 0.6125 μM of either **1** or **2**. b) Normalized lipoate reduction in A549 cells exposed to **1** or **2** across multiple concentrations. Error bars represent SEM.

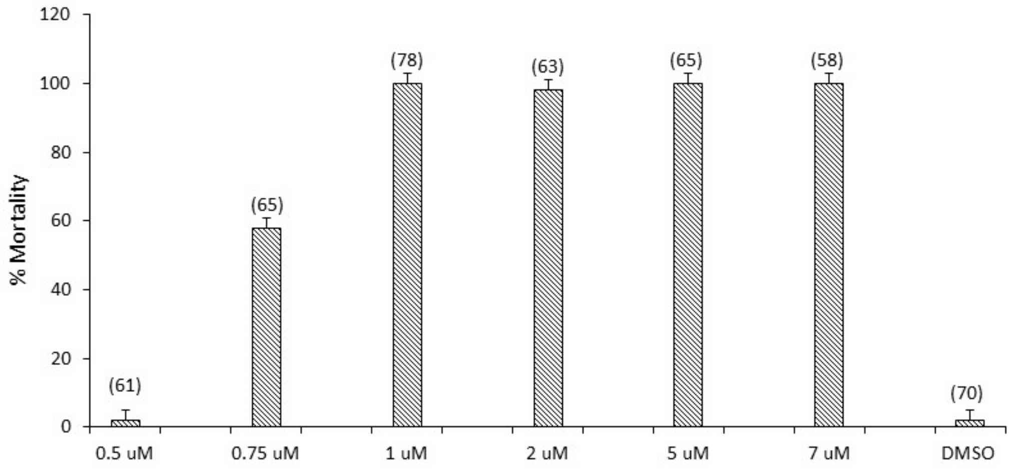

Complex **1**

Figure S29: **Toxicity analysis of Complex 1 in Zebrafish Larvae.** Two-day old zebrafish larvae were treated with various doses of Complex **1** and DMSO for 24-48 hours. After treatment, the larvae were analyzed for mortality and percent mortality for each treatment group is quantified and graphed. Each treatment was done three times and the total number of larvae treated per dose is indicated within parenthesis above the respective bars. The 0.75 μM dose of complex **1** induces ~50% mortality in the and is identified as the LD_{50} for zebrafish larvae. The 0.5 μM dose is identified as the Maximum Tolerated Dose (MTD). All these treatment groups are statistically significant from the control treatment ($p<0.001$, T-test).

References:

1. Sanderson, M. D.; Kamplain, J. W.; Bielawski, C. W., *J. Am. Chem. Soc.,* **2006,** *128* (51), 16514-16515.
2. Tennyson, A. G.; Lynch, V. M.; Bielawski, C. W., *J. Am. Chem. Soc.,* **2010,** *132* (27), 9420-9429.
3. Hashmi, A. S. K.; Hengst, T.; Lothschütz, C.; Rominger, F., *Adv. Synth. Catal.,* **2010,** *352* (8), 1315-1337.
4. Aranzaes, J. R.; Daniel, M.-C.; Astruc, D., *Can. J. Chem.,* **2006,** *84* (2), 288-299.
5. SAINT Bruker-AXS (2014b). SAINT Ver. 8.34A. Bruker AXS Inc., Madison, Wisconsin, USA.
6. Sheldrick, G., *Acta Crystallographica Section A,* **2008,** *64* (1), 112-122.
7. Sheldrick, G., *Acta Crystallographica Section A,* **2015,** *71* (1), 3-8.
8. Sheldrick, G., *Acta Crystallographica Section C,* **2015,** *71* (1), 3-8.
9. Sittaramane, V.; Padgett, J.; Salter, P.; Williams, A.; Luke, S.; McCall, R.; Arambula, J.F.; Graves, V.B.; Blocker, M.; Van Leuven, D.; Bowe, K.; Heimberger, J.; Cade, H.C.; Immaneni, S.; Shaikh, A., Chem. Med. Chem. **2015,** 10 (11), 1802-7.
10. He, H.; Li, X.; Feng, D.; Wu, M.; Chen, R.; Chen, T.; Chen, D.; Feng, X., *Chem. Res. Toxicol.* **2013**, *26*, 89.
11. Fako, V. E.; Furgeson, D. Y., *Adv. Drug Deliv. Rev.* **2009**, *61*, 478.
12. Hill, A. J.; Teraoka, H.; Heideman, W.; Peterson, R. E., *Toxicol. Sci.* **2005**, *86*, 6.
13. Zhang, J.; Qian, J.; Tong, J.; Zhang, D.; Hu, C., *Chem. Res. Toxicol.* **2013**, *26*, 1168.
14. Heimberger, J.; Cade, H. C.; Padgett, J.; Sittaramane, V.; Shaikh, A., *Bioorg. Med. Chem. Lett.* **2015**, *25*, 1191.
15. Westerfield, M., *The Zebrafish Book. A Guide for the Laboratory Use of Zebrafish* (Danio rerio), 4th ed.; University of Oregon Press: Eugene, **2000**.
